# Phylogenetic placement of the monotypic *Baolia* (Amaranthaceae s.l.) based on morphological and molecular evidence

**DOI:** 10.1186/s12870-024-05164-8

**Published:** 2024-05-25

**Authors:** Shuai Liu, Marie Claire Veranso-Libalah, Alexander P. Sukhorukov, Xuegang Sun, Maya V. Nilova, Maria Kushunina, Jannathan Mamut, Zhibin Wen

**Affiliations:** 1 College of Life Sciences, Xinjiang Agricultural University, Urumqi, 830052 China; 2 Biodiversität und Evolution der Pflanzen, Prinzessin Therese von Bayern-Lehrstuhl für Systematik, Ludwig-Maximilians-Universität München, Menzinger Str. 67, 830052 München, Germany; 3 Department of Higher Plants, Biological Faculty, Lomonosov Moscow State University, Moscow, 119234 Russian Federation; 4 Laboratory Herbarium (TK), Tomsk State University, Tomsk, 634050, Russian Federation; 5 College of Forestry, Gansu Agricultural University, Lanzhou, 730070 China; 6 Department of Plant Physiology, Biological Faculty, Lomonosov Moscow State University, Moscow, 119234 Russian Federation; 7 State Key Laboratory of Desert and Oasis Ecology, Key Laboratory of Ecological Safety and Sustainable Development in Arid Lands, Xinjiang Institute of Ecology and Geography, Chinese Academy of Sciences, Urumqi, 830011 China; 8 Xinjiang Key Lab of Conservation and Utilization of Plant Gene Resources, Urumqi, 830011 China; 9 Sino-Tajikistan Joint Laboratory for Conservation and Utilization of Biological Resources, Urumqi, 830011 China; 10 The Specimen Museum of Xinjiang Institute of Ecology and Geography, Chinese Academy of Sciences, Urumqi, 830011 China

**Keywords:** *Baolia*, Corispermoideae, Morphoanatomical character, Plastid genome, Chenopodiaceae

## Abstract

**Background:**

*Baolia* H.W.Kung & G.L.Chu is a monotypic genus only known in Diebu County, Gansu Province, China. Its systematic position is contradictory, and its morphoanatomical characters deviate from all other Chenopodiaceae. Recent study has regarded *Baolia* as a sister group to Corispermoideae. We therefore sequenced and compared the chloroplast genomes of this species, and resolved its phylogenetic position based on both chloroplast genomes and marker sequences.

**Results:**

We sequenced 18 chloroplast genomes of 16 samples from two populations of *Baolia bracteata* and two *Corispermum* species. These genomes of *Baolia* ranged in size from 152,499 to 152,508 bp. Simple sequence repeats (SSRs) were primarily located in the LSC region of *Baolia* chloroplast genomes, and most of them consisted of single nucleotide A/T repeat sequences. Notably, there were differences in the types and numbers of SSRs between the two populations of *B. bracteata*. Our phylogenetic analysis, based on both complete chloroplast genomes from 33 species and a combination of three markers (ITS, *rbcL*, and *matK*) from 91 species, revealed that *Baolia* and Corispermoideae (*Agriophyllum*, *Anthochlamys*, and *Corispermum*) form a well-supported clade and sister to *Acroglochin*. According to our molecular dating results, a major divergence event between *Acroglochin*, *Baolia*, and Corispermeae occurred during the Middle Eocene, approximately 44.49 mya. Ancestral state reconstruction analysis showed that *Baolia* exhibited symplesiomorphies with those found in core Corispermoideae characteristics including pericarp and seed coat.

**Conclusions:**

Comparing the chloroplast genomes of *B. bracteata* with those of eleven typical Chenopodioideae and Corispermoideae species, we observed a high overall similarity and a one notable noteworthy case of inversion of approximately 3,100 bp. of DNA segments only in two *Atriplex* and four *Chenopodium* species. We suggest that Corispermoideae should be considered in a broader sense, it includes Corispermeae (core Corispermoideae: *Agriophyllum*, *Anthochlamys*, and *Corispermum*), as well as two new monotypic tribes, Acroglochineae (*Acroglochin*) and Baolieae (*Baolia*).

**Supplementary Information:**

The online version contains supplementary material available at 10.1186/s12870-024-05164-8.

## Background

The family Chenopodiaceae (Amaranthaceae s.l.) with approximately 110 genera and 1700 species, is a large clade divided into seven subfamilies: Betoideae, Camphorosmoideae, Chenopodioideae, Corispermoideae, Salicornioideae, Salsoloideae, and Suaedoideae [1]. These major groups within Chenopodiaceae s.s. are distinguished by a set of morphoanatomical characteristics that make them visually distinguishable, as demonstrated by several studies [2–4]. Over the last two decades, numerous molecular phylogenetic studies have significantly enhanced our understanding of relationships within each subfamily. For example, Hohmann et al. [5] explored that Betoideae and included *Beta*, *Hablitzia*, *Patellifolia*, *Oreobliton*, and *Aphanisma*. The origins of the *Oreobliton* and *Aphanisma* species showed an evolution towards drier habitats. Kadereit and Freitag [6] examined the relationship between Camphorosmoideae and Salsoloideae, and provided a revised classification of Camphorosmoideae including Camphorosmeae, as well as descriptions of the new genera *Spirobassia*, *Eokochia*, *Grubovia* and *Sedobassia*. Fuentes-Bazan et al. [7, 8], Sukhorukov et al. [9], Uotila et al. [10] suggested that Chenopodioideae can be divided into Anserineae, Axyrideae, Dysphanieae, and Chenopodieae (incl. Atripliceae). Shepherd et al. [11] and Kadereit et al. [12] provided an insight into Salicornioideae and the relationships among the clade *Sarcocornia* + *Salicornia* (*Salicornia* s.l.) and especially the Australian members were clarified. Additionally, Akhani et al. [13] and Wen et al. [14] focused on Salsoloideae that greatly improved the phylogenetic position of their members dividing them into Salsoleae and Caroxyleae. Schütze et al. [15] studied Suaedoideae with further merger of *Alexandra* and *Borszczowia* into *Suaeda*.

A monotypic genus *Baolia* H.W.Kung & G.L.Chu, discovered only a few decades ago [16], remained enigmatic for a long time due to its limited distribution in Central China with only one collection from the type locality in Diebu [Têwo] county, Gansu province. Recently, *Baolia bracteata* H.W.Kung & G.L.Chu was rediscovered 15 km east from the type locality and included in a phylogenetic analysis using nuclear (nrITS) and two chloroplast markers (*rbcL* and *atpB*-*rbcL*) [17]. This analysis resolved it as a sister group to Corispermoideae, which includes *Corispermum* L., *Agriophyllum* M.Bieb., and *Anthochlamys* Fenzl [17]. Despite their close phylogenetic positions, *Baolia* and Corispermoideae exhibit high heterogeneity in morphological characteristics [16, 18, 19].

Unlike gene fragments, complete chloroplast genomes encompass a greater amount of genetic information and mutation sites. These attributes prove advantageous in various aspects including phylogenetic analysis, assessment of genetic diversity, and plant molecular identification [20, 21]. Until now, chloroplast genomes from only a limited number of Chenopodiaceae species have been deposited in GenBank (https://www.ncbi.nlm.nih.gov/sra). However, numerous genera within the family still lack representation, and the prospect of establishing a comprehensive phylogeny based on complete plastomes of Chenopodiaceae s.s. remains a distant goal. To address this issue, a solution lies in leveraging the multitude of sequences amassed from molecular phylogenetic investigations of Chenopodiaceae over the years, which could provide a more comprehensive and in-depth sampling.

Consequently, this study aims to generate new sequences (nuclear ribosomal ITS and two plastid loci *rbcL* and *matK*) to complement available GenBank sequences and resolve phylogenetic relationships between *Baolia* and closely related taxa. Furthermore, the placement of *Acroglochin* warrants thorough discussion. In a recent study [17], this genus was found to be a sister to the ‘*Baolia* + Corispermoideae’ clade. Considering the previously proposed phylogenetic position of *Acroglochin* either within Betoideae [1] or in close proximity to *Corispermum* [5, 22, 23], a reevaluation becomes imperative.

Using new and previously generated molecular data, our objectives were as follows: (1) to scrutinize variations in the structure and composition of chloroplast genomes in two *Baolia* populations, while conducting a comparative analysis with eleven typical Chenopodioideae and Corispermoideae species; (2) to elucidate the phylogenetic relationships between *Acroglochin*, *Baolia*, and Corispermoideae; (3) to evaluate and reconstruct ancestral states of significant morphoanatomical traits.

## Results

### Genome structural variation

The chloroplast genome of *Baolia bracteata* exhibited the typical tetrad cyclic structure, comprised of the LSC (86,140 − 86,146 bp), SSC (20,118 − 20,127 bp) and two IR regions (23,118 bp) (Additional file 1: Fig. S1). The length of the plastid genome ranged from 152,499 to 152,508 bp (Additional file 2: Table S1). In contrast to the LSC and SSC regions, the variation in length of the IR region was relatively small. The GC content of the LSC, SSR, IRs was 36.7%, 34.5%, 30.7%, and 43.4%, respectively. The distribution of GC content was uneven throughout the whole chloroplast genome (Additional file 2: Table S1). The chloroplast genome comprised a total of 131 genes, including 86 protein-coding genes, 38 tRNA genes, and 8 rRNA genes. Additionally, *rpl23* and *rps19* were identified as pseudogenes (Additional file 2: Table S2). Among these 131 genes, 14 contained one intron (*atpF*, *ndhA*, *ndhB*, *petB*, *petD*, *rpl2*, *rpl16*, *rpoC1*, *trnI-GAU*, *trnG-GCC*, *trnL-UAA*, *trnV-UAC*, *trnA-UGC*, and *trnK-UUU*), while 3 contained two introns (*rps12*, *ycf3*, and *clpP*) (Additional file 2: Table S3).

Chloroplast genome sizes of eleven analyzed species from Chenopodioideae and Corispermoideae ranged from 150,590 to 152,237 bp. *Atriplex centralasiatica* Iljin had the largest plastome size while *Corispermum declinatum* Stephan ex Iljin had the smallest plastome size. The total number of genes among these eleven species ranged from 129 to 133, encompassing 84–88 protein coding genes, 37 tRNA genes and 8 rRNA genes. The total GC content of these chloroplast genomes (36.9–37.3%), LSC regions (34.7–35.4%), SSC regions (30.3–31.0%), and IR regions (42.7–42.8%) did not exhibit significant differences across different species (Additional file 2: Table S1).

### Simple repetitive sequences (SSRs) and repetitive sequences

**Fig. 1:**
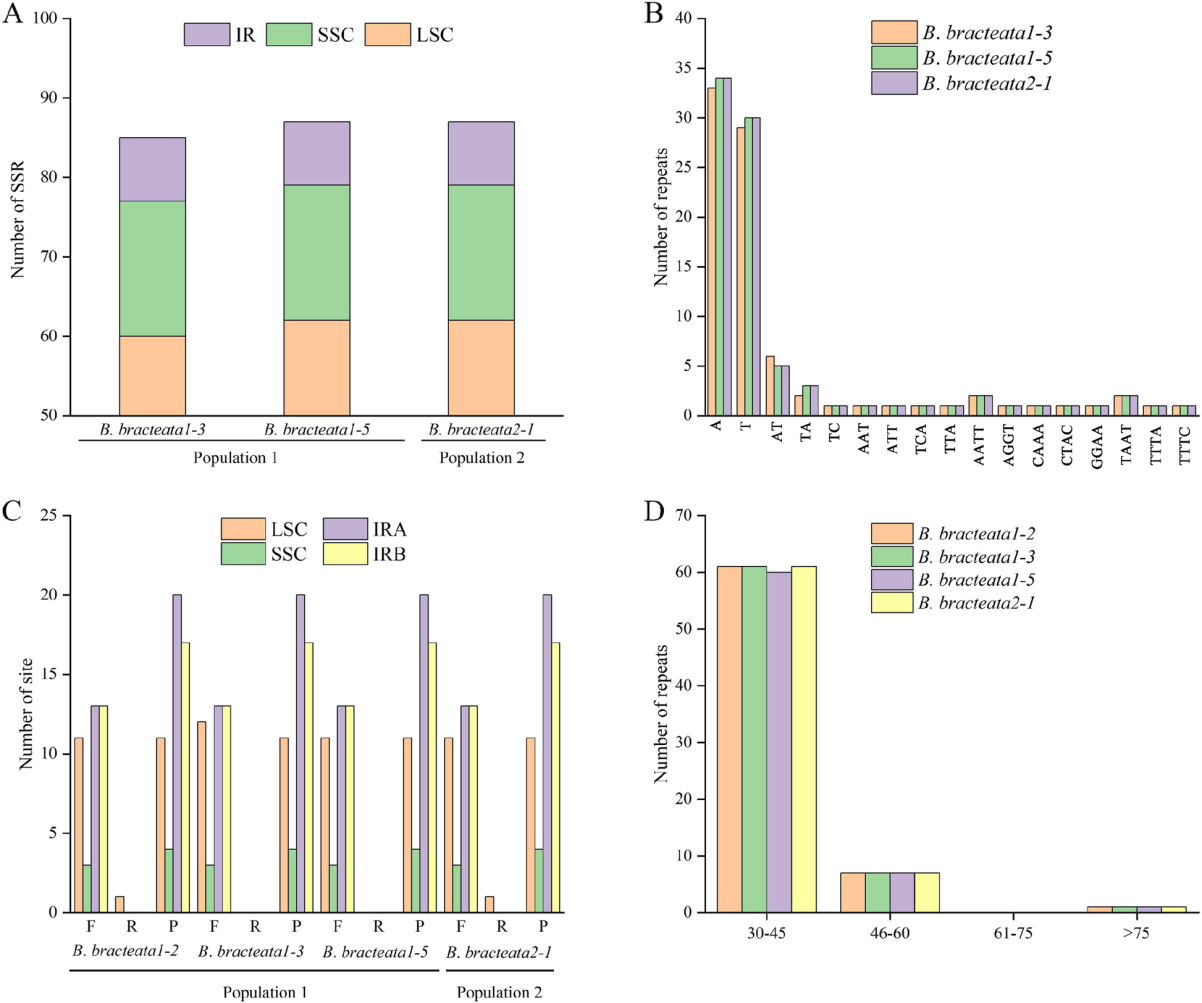
Types and distributions of repeat sequences and short sequence repeats (SSRs) in *Baolia bracteata* chloroplast genomes. **A** The number of SSR loci in different chloroplast genome regions. **B** Distribution of repeats classified by type. **C** Number and position repeat sequences in four *B. bracteata* chloroplast genomes. **D** The length of the plastid repeat sequence in *B. bracteata*

A total of 1,386 SSRs were identified in the 16 chloroplast genomes of the two populations of *Baolia bracteata*. To analyze the characteristics of these SSRs, we selected three types with different numbers of SSRs: *B. bracteata* 1–3 and 1–5 in population 1, and *B. bracteata* 2 − 1 in population 2. This selection allowed further investigation of the type and distribution of SSRs (Fig. 1A). Of the total of SSRs, 70.59–71.26% were located in the LSC region, 9.20–13.30% in the IR region and 19.54-20.00% in the SSC region. Notably, *B. bracteata* 1–3 had two fewer A and T single-nucleotide repeats compared to *B. bracteata* 1–5 and 2 − 1. These repeats were found in the LSC, IGS (*ycf4, cemA*) and the *rpl*16-intron1 (Fig. 1B; Additional file 2: Table S4). Importantly, approximately 29-73.56% of the total of SSRs consisted of A/T single-nucleotide repeat sequences, suggesting an A/T nucleotide bias among the chloroplast SSRs of *B. bracteata*.

To characterize the *B. bracteata* chloroplast genome, we analyzed four types of repeat sequences: forward repeats (F), reverse repeats (R), palindromic repeats (P), and complementary repeats (C). All four types of repetitive sequences were detected in *B. bracteata* 1–2, 1–3, 1–5 in population 1, and *B. bracteata* 2 − 1 in population 2. These representative types were then used to study the positions of these repeats. We found 68–69 repetitive sequences (> 10 bp), including one R-type repetition, 34 P-type repetitions, and 33–34 F-type repetitions. However, C-type repetition was not identified. Most of the forward and palindromic repeats, as well as all the reverse and complementary repeats, were located in the LSC region. Both *B. bracteata* 1–2 and 2 − 1 exhibited an R-type repetition, located in the *rpl16*-intron1 gene within the LSC region. *Baolia bracteata* 1–2, 1–5, and 2 − 1 displayed 11 F-type repetitions. In contrast, *B. bracteata*1-3 had 12 F-type repetitions. Notably, *B. bracteata* 1–3 contained an additional F-type repeat sequence situated in *ycf3*-intron1 or IGS (*rps*12, *trnV*-*GAC*), distinguishing it from *B. bracteata* 1–2, 1–5, and 2 − 1 (Fig. 1C; Additional file 2: Table S4).

The lengths of the repeats ranged from 30 to 30,118 bp. Based on their length, we categorized the repeats into four categories: 30–45 bp, 45–60 bp, 60–75 bp, and > 75 bp. The majority of repeats (88.24–88.40%) were within the 30–45 bp range, while 10.14–10.29% fell within 46–60 bp range, and 1.45–1.50% exceeded 75 bp in length (Fig. 1D; Additional file 2: Table S4).

### Comparative genomic analysis

**Fig. 2:**
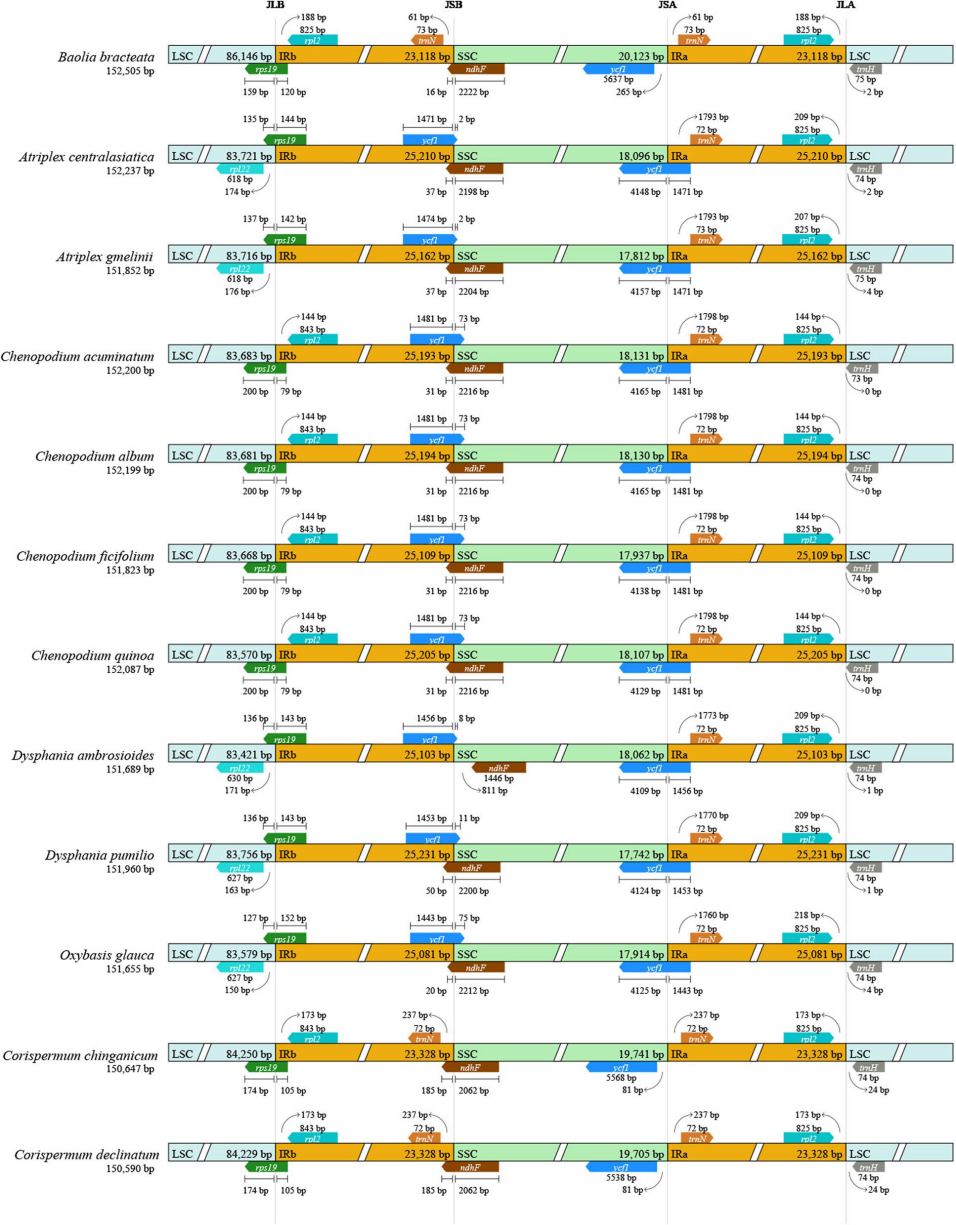
The borders of large single copy (LSC), small single copy (SSC), and inverted repeat (IR) regions among 12 chloroplast genomes. The number above the gene features means the distance between the ends of genes and the borders sites

Using *Baolia bracteata* as a reference, we conducted an analysis of the junction sites between the IR and SC regions in comparison with eleven species from Chenopodioideae and Corispermoideae (Fig. 2). The sizes of the IR region ranged from 23,118 to 25,231 bp, encompassing the *rpl2* and *trnN* genes, while the LSC region contained the *rpl22* and *trnH* genes. In most species, the SSC/IRb boundary was situated in the coding regions of the *ycf1* and *ndhF* genes. However, in *B. bracteata*, *Corispermum chinganicum* Iljin and *C. declinatum*, the SSC/IRb boundaries of were located exclusively in the *ndhF* gene. Similarly, for *Dysphania ambrosioides* (L.) Mosyakin & Clemants, this boundary was found only within the *ycf1* gene. The junction of the LSC/IRb region contained the *rps19* gene. The IRa/SSC boundary was identified within the *ycf1* gene, with *B. bracteata*, *C. chinganicum*, and *C. declinatum* exhibiting a complete IRa/SSC boundary of the *ycf1* gene within the SSC region (Fig. 2).

Furthermore, we conducted a comprehensive sequence mVISTA homology analysis of the chloroplast genomes of these 12 species (Additional file 1: Fig. S2). These genomes exhibited similarities in terms of length, structure and gene distribution. A high degree of homology was observed across all genomes, with a few regions displaying less than 90% homology. Notably, the IR region demonstrated greater conservation than the SC region, and coding regions exhibited higher conservation compared to non-coding regions. The multiple comparison analysis using Mauve revealed substantial interlocking blocks within the chloroplast genomes of all 12 species. However, a notable inversion of approximately 3,100 bp was observed at the LSC position in two *Atriplex* L. and four *Chenopodium* L. species, containing the genes *rbcL*-*atpB*-*atpE*-*trnM*-*trnV* (Additional file 1: Fig. S3).

### Phylogenetic analysis

**Fig. 3:**
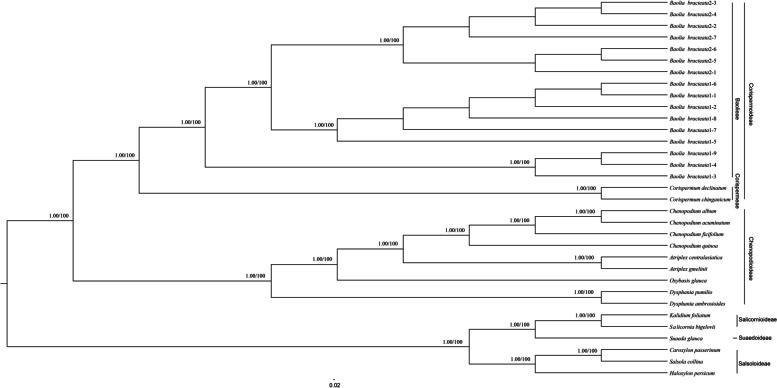
Phylogenetic tree reconstruction of the 33 species inferred from Maximum Likelihood (ML) and Bayesian Inference (BI) analyses based on the complete plastomes. Bayesian posterior probabilities / ML bootstrap values are shown above branches. Branches with support rates of not 100% and 1 are not marked

For the phylogenetic analysis, we utilized 33 complete chloroplast genome sequences from 18 species, comprising 18 newly sequenced chloroplast genomes from *C. chinganicum*, *C. declinatum* and *B. bracteata*, as well as sequences for 15 species downloaded from NCBI (Additional file 2: Table S5). The maximum likelihood (ML) and Bayesian inference (BI) methods were employed to generate phylogenetic trees, both of which yielded congruent topologies. Specifically, all *B. bracteata* samples formed a well-supported monophyletic clade (bootstrap support, bs = 100%; posterior probability, pP = 1). This clade was identified as the sister group to two *Corispermum* species (bs = 100%; pP = 1). Species from Chenopodioideae including *Chenopodium*, *Atriplex*, *Oxybasis*, and *Dysphania* collectively formed a well-supported clade (bs = 100%; pP = 1) (Fig. 3).

For marker sequences employed in the phylogenetic analysis (ITS, *rbcL*, and *matK*), a dataset of 236 sequences, which included 18 newly obtained sequences, representing 91 species, was used (Additional file 2: Table S6). The concatenated data matrix encompassed 3,665 characters. The ML analysis conducted on the three genes resulted in an optimal single tree (-ln*L* = 37562.0677). In particular, a monophyletic group comprising six representative *B. bracteata* samples was identified (bs = 100%; pP = 1). This monophyletic group emerged as the sister clade to Corispermeae (bs = 100%, pP = 1) (Additional file 1: Fig. S4). Additionally, *Acroglochin* was resolved as the sister taxon to the clade comprising Corispermoideae (*Agriophyllum*, *Anthochlamys*, and *Corispermum*) and the *Baolia* clade (bs = 87%, pP = 1).

### Dated molecular phylogeny

For divergence time estimation, our analysis focused exclusively on Corispermoideae and Chenopodioideae along with the genera *Acroglochin* and *Baolia*. The *rbcL* and *matK* matrix comprised 2,879 characters and 43 species, and the ITS matrix comprised 684 characters and 45 species. The divergence trees resulting from the *matK* + *rbcL* datasets is presented in Additional file 1: Fig. S5. Within this tree, Corispermeae and *Baolia* formed a monophyletic clade that was a sister to *Acroglochin*. The divergence tree resulting from the ITS is shown in Additional file 1: Fig. S6. In this tree, Corispermeae and *Acroglochin* formed a weakly supported clade sister to *Baolia*. Based on the outcomes of molecular dating, a major split between *Acroglochin*, *Baolia*, and Corispermeae occurred during the Middle Eocene approximately 44.49 (59.40–27.33) mya. Additional dates for various lineages can be found in Table 1.

**Table 1:** Results of the divergence time estimates (in Ma) calculated with program BEAST

Node	matK + rbcL gene	ITS	Geological epoch
Crown age of Chenopodioideae	56 (57.97–54.05)	56.06 (57.99–54.11)	Early Eocene
Split between Corispermeae / Baolieae and Acroglochineae	44.49 (59.40–27.33)	- -	Middle Eocene
Stem age of Baolieae	31.29 (47.52–17.48)	40.88 (59.27–21.54)	Late Eocene-Early Oligocene
Crown age of Baolieae	1.5 (3.51–0.33)	1.86 (4.78–0.38)	Middle Pliocene-Middle Pleistocene
Crown age of Corispermeae	21.23 (34.35–10.81)	17.84 (32.4–6.8)	Early-Middle Miocene
Crown age of Acroglochineae	0.42 (2.2–0.01)	0.67 (2.7–0.01)	Middle-Late Pleistocene

Numbers given in brackets represent 95% confidence intervals

### Fruit and seed anatomy of *Baolia* and *Acroglochin*

#### Baolia

**Fig. 4:**
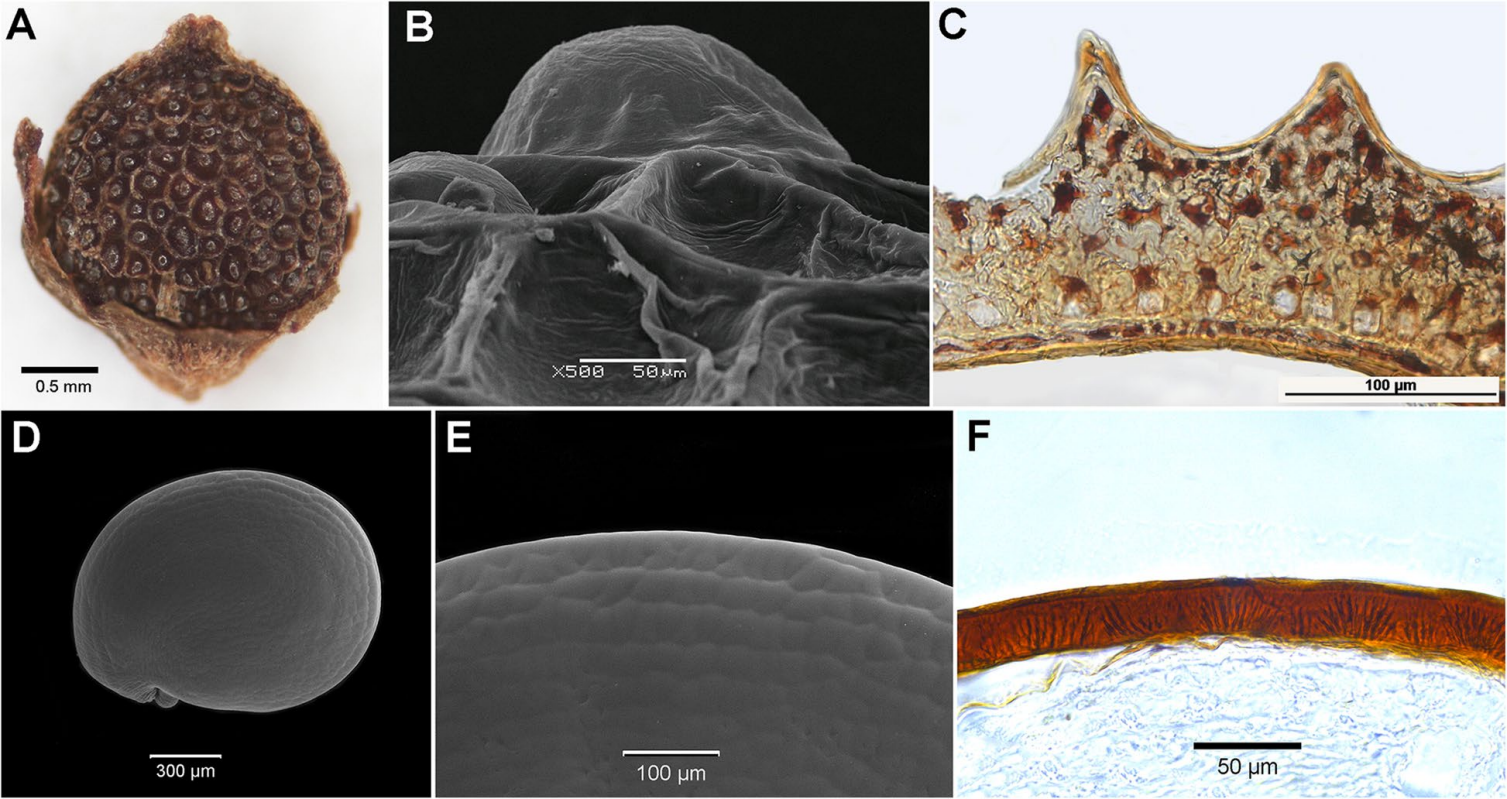
Fruit anatomy of *Baolia bracteata* (**A** *-* **C**) and *Acroglochin persicarioides* (**D** *-* **F**): (**A**) - fruit of *B. bracteata*; (**B**) - foveolate fruit surface of *B. bracteata*; (**C**) - cross-section of pericarp and seed-coat of *B. bracteata*; (**D**) - seed of *A. persicarioides*; (**E**) - seed surface of *A. persicarioides*; (**F**) - cross-section of *A. persicarioides* seed coat

The fruit is indehiscent and displays a distinctive foveolate surface, setting it apart from other members of Chenopodiaceae s.s. Our investigation has revealed that these foveolae are a result of the bursting or compression of the outer walls of the exocarp cells during the drying process that follows fruit ripening (Fig. 4A). Upon soaking, many exocarp cells regain their original mamillate shape (Fig. 4B). The mesocarp (Fig. 4C) consists of brachysclereids, characterized by small lumens filled with brown tannin-like substances. This supportive tissue contributes to the fruit’s firmness. The lowermost layers of the mesocarp contain monoprismatic crystals. The endocarp is composed of a single layer with thickened cell walls. The seed coat is superficially smooth, closely attached to the pericarp but not fused with it. It is thin, comprising two compressed layers, with tannin-filled cells. Occasionally, one to several colorless intermediate layers can be observed between these layers. Perisperm is abundant, and the embryo is annular and positioned vertically.

#### Acroglochin

The fruit is one-seeded, dehiscent through a lid. The pericarp exhibits a greenish hue and consists of multiple parenchymatous layers. The seeds are dark-red, somewhat depressedly-roundish, or slightly elongated, with a shiny surface with marginal keeling and polygonal cell shape (Fig. 4D, E). The seed-coat testa measures 25–30 μm in thickness and features stalactite-like formations in the outer cell walls (Fig. 4F). The tegmen is significantly thinner, made up of 2–3 compressed cell layers. Perisperm is abundant, and the embryo is annular and positioned vertically.

### Ancestral state reconstruction

**Fig. 5:**
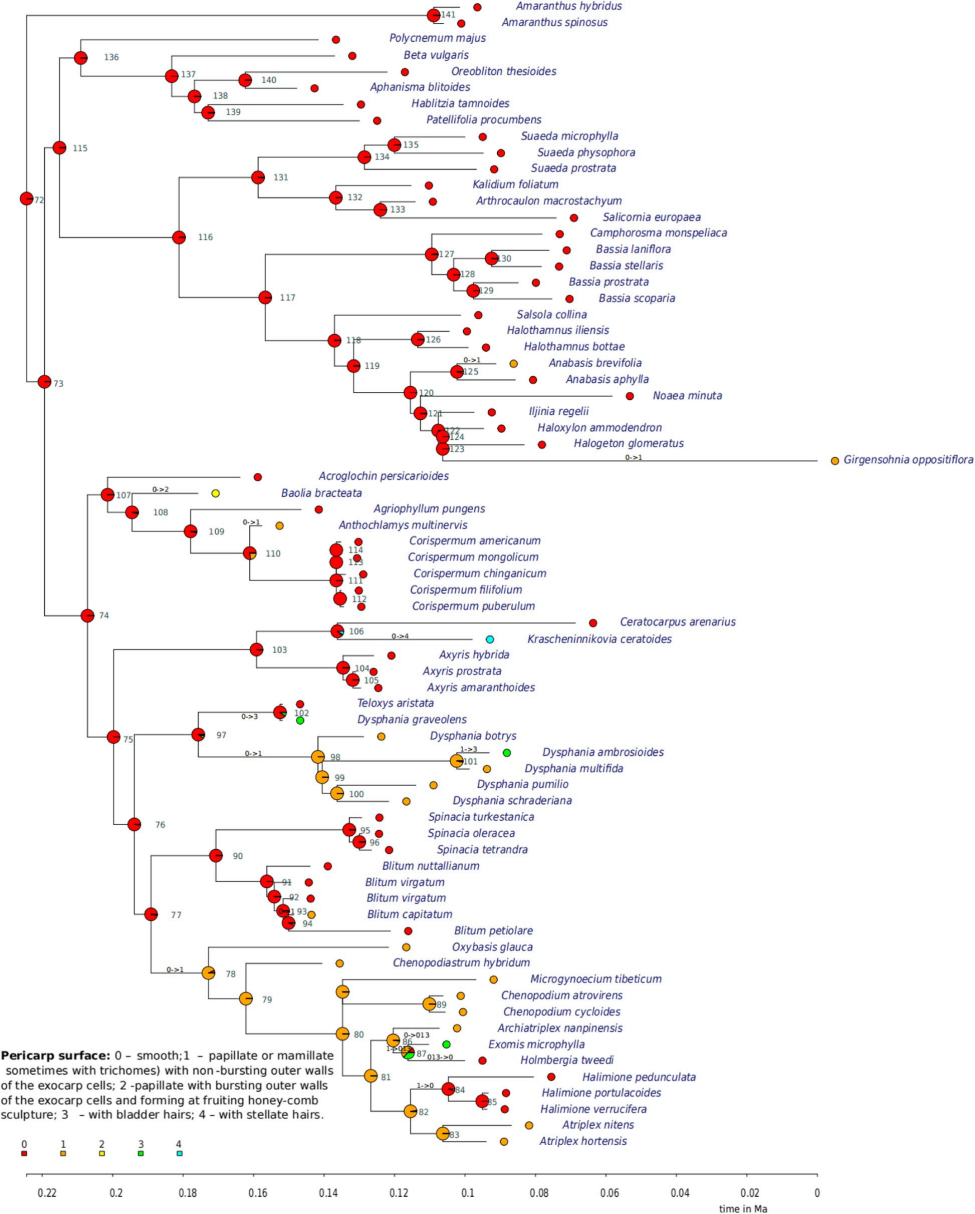
Ancestral character reconstruction of pericarp surface characters in Corispermoideae. Pericarp surface: 0 - smooth; 1 - papillate or mamillate (sometimes with trichomes) with non-bursting outer walls of the exocarp cells; 2 - papillate with bursting outer walls of the exocarp cells and forming at fruiting honey-comb sculpture; 3 - with bladder hairs; 4 - with stellate hairs

The ancestral state reconstruction revealed that characters formerly employed for defining *Baolia*, *Acroglochin*, and Corispermoideae exhibit varying degrees of homoplasy (Table 2; Additional file 2: Tables S7-S17; Additional file 1: Figs. S7-S15). For instance, attributes such as fruit dehiscence, the presence of sclerenchymatous tissue in the pericarp, and the thickness of the seed coat testa display complex patterns of convergence (Fig. 5; Additional file 1: Fig. S15). Notably, the presence of acicular apices appears to be an apomorphic state shared between *Acroglochin* and *Teloxys* (Additional file 1: Fig. S8). A similar pattern emerges for the inflorescence structure featuring clusters of monochasium, which represents a derived state in *Acroglochin*, *Ceratocarpus*, and *Teloxys* (Additional file 1: Fig. S9).

Several traits within *Baolia* exhibit symplesiomorphies with those found in core Corispermoideae (*Agriophyllum*, *Anthochlamys*, and *Corispermum*), including characteristics like seed-coat testa, pericarp with sclerenchymatous tissue (Fig. 4C; Additional file 1: Figs. S14-S15). A noteworthy apomorphy in *Baolia* involves papillate fruits with honeycomb-like surface formed by ruptured outer walls of exocarp cells (Figs. 4A and C and 5). The current ancestral character reconstruction underscores the necessity for a meticulous reassessment of the morphological attributes that have traditionally been employed in delineating the boundaries of *Baolia* and *Acroglochin*.

**Table 2:** Results of the ancestral character state analysis on *Baolia*, *Acroglochin* and core Corispermoideae genera

Character number	state	Reconstructed ancestral state (crown node 73)	Number of character state shifts	Figure
1.	Hairs on stems and leaves	Absent or papillae	13 (three shifts from no hairs or papillae to mostly simple curved hairs; one shift from no hairs or papillae to dendroid hairs; one shift from simple curved hairs to branched hairs; one shift from no hairs or papillae to prevailing stellate hairs; one shift from no hairs or papillae to three to five hyaline segments within one individuum; one shift from no hairs or papillae & of two accrescent segments forming bract-like cover and five green segments to dendroid hairs); 4 (three reversals from dendroid hairs to no hairs or papillae; one reversal from simple, glandular hairs and subsessile glands to no hairs or papillae)	S7
2.	Acicular apices	Acicular apices absent	2 (shifts from no acicular apices to acicular apices)	S8
3.	Inflorescence	Composed of clusters	7 (four shifts from composed of clusters to spikes; 3 shifts from composed of clusters to monochasium)	S9
4.	Bracteoles	Absent	7 (seven shifts from no bracteoles to bracteoles)	S10
5.	Perianth	Absent or reduced (1–2 segments) and hyaline	9 (five shifts from absent or reduced (1–2 segments) and hyaline to five hyaline segments; three shifts from absent or reduced (1–2 segments) and hyaline to five hyaline segments to three or four green segments usually [turning] fleshy; one shift from absent or reduced (1–2 segments) and hyaline to five hyaline segments (4)5 green segments; and one reversal shift from five hyaline segments to absent or reduced (1–2 segments) and hyaline	S11
6.	Fruit	Indehiscent	8 (four shifts from indehiscent to dehiscent by a lid; 4 shifts from indehiscent to dehiscent by a lid)	S12
7.	Pericarp surface	Papillate or mamillate (sometimes with trichomes) with non-bursting outer walls of the exocarp cells	11 (six shifts from smooth pericarp surface to papillate or mamillate (sometimes with trichomes) with non-bursting outer walls of the exocarp cells; 2 papillate or mamillate (sometimes with trichomes) with non-bursting outer walls of the exocarp cells to bladder hairs; one shift from smooth to with bladder hairs: 1 shift from smooth pericarp surface to surface with two accrescent segments forming bract-like cover and five green segments; 1 shift from smooth pericarp surface to papillate with bursting outer walls of the exocarp cells and forming at fruiting honey-comb sculpture; one reversal from papillate or mamillate (sometimes with trichomes) with non-bursting outer walls of the exocarp cells to smooth pericarp surface	5
8.	Pericarp wing	Absent	One shift from no pericarp wing to marginally present	S13
9.	Sclerenchymatous tissue in the pericarp	No sclerenchymatous tissue in the pericarp	4 (four shifts from sclerenchymatous tissue in the pericarp absent to present; one reversal from sclerenchymatous tissue in the pericarp present to absent)	S14
10.	Seed-coat testa	Seed coat more than 10 μm	Three shifts from seed coats of more than 10 μm to both very thin (up to 8 μm) and more than 10 μm); 6 shifts from seed coat more than 10 μm to very thin (up to 8 μm)	S15

## Discussion

### Repetitive sequence, comparative genomic analysis and phylogenetic inference

Repetitive sequences within chloroplast genomes offer valuable insights into genome rearrangements, sequence divergence, and can serve as useful molecular markers for phylogenetic and population studies [24, 25]. An analysis of the chloroplast genomes of 16 *B. bracteata* samples from two populations revealed the presence of 85 to 87 SSRs (Fig. 1A). These SSRs were predominantly located in the LSC region, and the majority of them consist of single-nucleotide A/T repeat sequences, a pattern consistent with the chloroplast genomes of most angiosperms [26, 27], and they contributed significantly to the A/T abundance of the plastid genome. The abundance of long repeats and SSRs in the intergenic region (Additional file 2: Table S4) may result from variants, including indels and SNPs [28, 29]. The variations in SSR types and numbers, as well as repetitive sequences, between the two populations of *B. bracteata* were distinct (Fig. 1), providing valuable insights for further studies on the level of population genetic diversity.

In most plants, the boundaries and junctions of the four structure parts of the chloroplast genome structure are conserved (e.g [21, 26–34]). Our results based on complete chloroplast genome analysis indicate that *B. bracteata* and eleven other species from Chenopodioideae and Corispermoideae exhibit highly conserved structure, gene content and gene order, with little variation between species. One notable exception is the presence of an inversion event in *Atriplex* and *Chenopodium* species (Additional file 1: Fig. S3). A previous study [35] detected sequence inversions in the *rbcL*-*trnV* region (~ 3.1 kb) of the chloroplast genomes of *Chenopodium quinoa* and *C*. *album* (Chenopodioideae). Large inversions have also been found in other taxa, like *Hevea brasiliensis* [36], *Annona cherimola* [37], *Viscum minimum* [38], *Passiflora edulis* [39]. These findings suggest that large inversions are relatively common in plant genomes. AT-rich regions are prone to inversion of large segments, but this phenomenon is not present in the three *Cypripedium* species with long inversions [40]; therefore, the relationship between inversions and AT-rich sequences remains uncertain.

In the previous study based on 48 species and three loci sequence data (ITS, *atpB*-*rbcL*, and *rbcL*), *Acroglochin* was resolved as sister to *Baolia* + Corispermeae and consequently considered part of the expanded Chenopodioideae [17]. In this study, the phylogenetic relationships of *Baolia* and Corispermoideae were resolved not only based on three loci sequence data (236 sequences from 91 species) but also on chloroplast genomes (33 accessions from 18 species). In both analyses, *B. bracteata* samples formed a well-supported clade, which was sister to core Corispermoideae (Fig. 3; Additional file 1: Fig. S4). *Acroglochin* was the sister taxon to the core Corispermoideae + *Baolia* clade (bs = 87%, pP = 1) (Additional file 1: Fig. S4). Species from Chenopodioideae formed a well-supported clade (bs = 100%, pP = 1), and they were sister to the clade composed of *Acroglochin*, core Corispermoideae, and *Baolia* (bs = 100%, pP = 1) (Additional file 1: Fig. S4).

### Geographical and spatial diversification of *Acroglochin* and *Baolia*

**Fig. 6:**
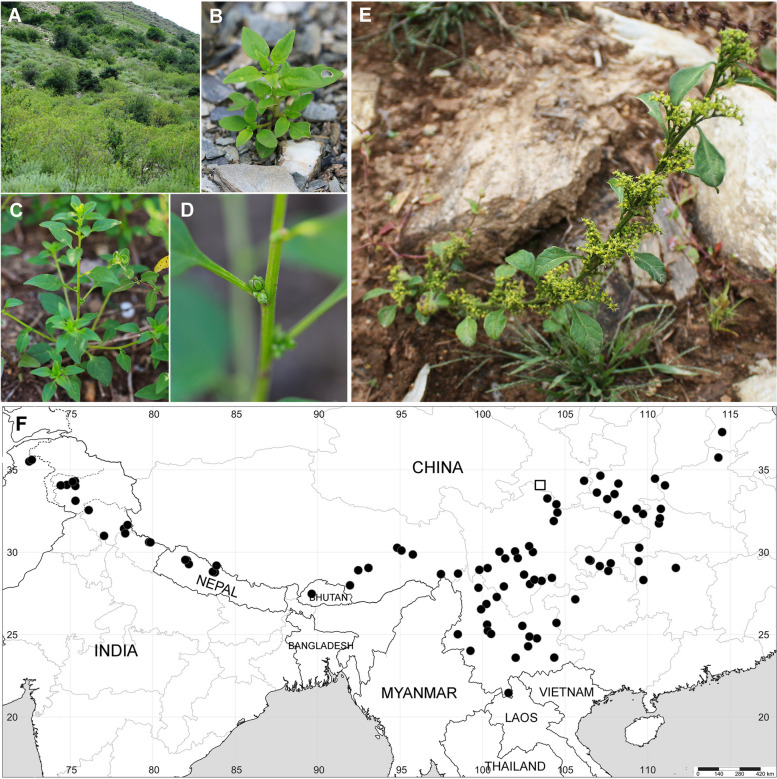
Geographical distribution, habitat and characteristics of *Baolia bracteata* and *Acroglochin persicarioides*. *Baolia bracteata* habitat and features: (**A**) - general view of the habitat; (**B**) - young plant; (**C**) - mature plant with flowers and fruits; (**D**) - close-up of the inflorescence. Photographs by Sun Xuegang (2021, Diebu [Têwo] County, Gansu Province, China). (**E**) - *(A) persicarioides* plant at fruiting stage. Photograph by Alexander Sukhorukov (September 2013, Mid-West Nepal). (**F**) - geographic distribution map of *(B) bracteata* (labeled by box) and *A. persicarioides* (labeled by circles). Multi-year provincial administrative boundary data in China from Resource and Environmental Sciences Data Registry and Publishing System, 2023 (http://www.resdc.cn/DOI). The labeled distribution loci in the figure are plotted by Maria Kushunina based on the distribution information of specimens seen

*Acroglochin* is indeed a remarkable genus within the Chenopodiaceae family. It exhibits two rare synapomorphies shared with unrelated members of Chenopodiaceae s.s. These characteristics include acicular apices terminating short branches (Fig. 6E), a trait it shares with *Dysphania tibetica* and *Teloxys aristata* (both belonging to the Dysphanieae tribe in Chenopodioideae), as well as a circumscissile fruit type, a feature found in many Betoideae members. These shared traits led to the initial classification of *Acroglochin* within the Betoideae subfamily (e.g [2, 5]).

However, recent phylogenetic data [17] have suggested that *Acroglochin* should be excluded from the Betoideae subfamily. As a result, the revised circumscription of Betoideae, which excludes *Acroglochin*, indicates that this subfamily is primarily found in regions such as the Mediterranean area, Macaronesia, West Europe, Asia Minor, and the Caucasus. The subfamily is also represented in the California floristic province of North America by the monotypic genus *Aphanisma* Nutt. With the exclusion of *Acroglochin* from Betoideae, the Himalaya and Tibet regions do not have any native representatives of Betoideae.

The number of species within *Acroglochin* has been a subject of taxonomic debate. Some earlier authorities [2, 5, 19] accepted only one species, *A. persicarioides* (Poir.) Moq. However, Zhu & Sanderson [30] recognized four species within *Acroglochin*, all of which were described from the Sichuan province of China. *Acroglochin* is known to have a wide distribution, including Bhutan, South/Central China, North India, Nepal, and North Pakistan (Fig. 6F). Records of *Acroglochin* may be found in northern Myanmar, Vietnam, and Laos. Typically, *Acroglochin* is found at elevations between 1700 and 3200 m above sea level, with its main distribution range in subtropical monsoon climates. There are a few records in the Tibetan Autonomous Region (Xizang province, China), primarily associated with higher altitudes that exceed the typical altitudinal range of *Acroglochin*.

In contrast, the distribution of the monotypic genus *Baolia* is confined to the vicinity of Diebu [Têwo] county in Gansu province, China (Fig. 6F) and only one collection from the type locality in Diebu was ever found. A new population (33˚56’47″N, 103˚44’15″E) found by one the authors (Sun Xuegang) was subsequently rediscovered 15 km to the east of the *Baolia* type locality. *Baolia* predominantly thrives on sunlit slopes in steppe habitats (Fig. 6A-D) at an elevation of approximately 1900 meters above sea level [16, 30]. These areas receive sufficient precipitation during the warm season. However, it’s important to note that the type locality of *B. bracteata* [16] faced a significant decline in population growth in the early 2000s due to escalating human activities, particularly related to new construction and changes in land use [41, 42]. Given its restricted range and habitat threats, *Baolia* should be classified as ‘Critically Endangered’ (CR) according to the IUCN Red List Categories [43].

In contrast, all members of the core Corispermoideae (including *Agriophyllum*, *Anthochlamys*, and *Corispermum*) exhibit a wide distribution across temperate, mostly (semi-) arid regions of Eurasia, with a few *Corispermum* species extending into North America. Some Corispermoideae species (*Agriophyllum tibeticum* Sukhor., *Corispermum* sp. div.) are also present in mountainous regions of Tibet, although they are typically found at much higher elevations ranging from 3000 to 5000 m above sea level [18, 19]. It is worth noting that none of the core Corispermoideae species are adapted to monsoon climates. As demonstrated here, the segregation of *Acroglochin*, *Baolia*, and core Corispermoideae is primarily driven by geographic and ecological divergence.

### Are there similarities between Baolia and Corispermoideae?

The morphological data do not provide strong evidence for close relationships between *Baolia* and all Corispermoideae. Several gross morphological characters shared by *Baolia* and Corispermoideae, such as the absence of acicular apices (character state 2:0) (Additional file 1: Fig. S8) and indehiscent fruits (6:0) (Additional file 1: Fig. S12), are common features found in nearly all members of the Chenopodiaceae. Among the micromorphological characters, carpological traits in Chenopodiaceae have been studied in great detail, revealing their taxonomic, evolutionary, and ecological implications [9, 11, 44–49]. The following fruit and seed characters appear to unite *Baolia* and Corispermoideae: (1) multi-layered pericarp with supporting tissue (character state 9:1) (Additional file 1: Fig. S14); (2) presence of tannin-like substances in the cell lumens; (3) thin seed coat consisting of two equal layers filled with tannins (character state 10:0) (Additional file 1: Fig. S15).

However, there are some distinctions between these characters in *Baolia* and Corispermoideae. For instance, in Corispermoideae, the supporting tissue in the pericarp is typically represented by fibers (brachysclereids are absent), and the presence of monocrystals in the pericarp has not been detected [18, 44].

These subtle differences in carpological traits suggest that while *Baolia* and core Corispermoideae share some micromorphological features, they also exhibit distinct characteristics, further complicating their taxonomic relationships based solely on morphology.

### Taxonomic treatment

We propose to consider the subfamily Corispermoideae Raf. in a broader sense, including the tribe Corispermeae (*Corispermum*, *Anthochlamys*, and *Agriophyllum*), and to describe two additional tribes, Acroglochineae and Baolieae. In the recent circumscription, the subfamily is very heterogeneous. An improved description of the tribe Corispermeae (or core Corispermoideae) was provided by Sukhorukov [18, 44].

Acroglochineae Sukhor. & Z.-B.Wen, trib. nov.

Type: *Acroglochin* Schrad. in Roem. & Schult.

Annuals, glabrous or scarcely pubescent with simple hairs, branches terminating in acicular apices. Leaves alternate, long-petiolate, broadly ovate or ovoid, dentate or erose, teeth straight or incurved, tip mucronate. Inflorescences leafy, monochasial, falsely dichotomous. Flowers bisexual. Perianth of 5 free segments, keeled along midrib. Stamens 2, anthers small, without appendages. Stylodia 2, concrescent into a style in their lower half. Fruit dehiscent by a lid; pericarp smooth, white or greenish with several homocellular layers. Seeds dark-red, depressedly-globular, ~ 1.3 mm in diameter, smooth, with crustaceous testal layer; embryo horizontal.

One to four species in Himalaya, Central and South China.

Baolieae Sukhor. & Z.-B.Wen, trib. nov.

Type: *Baolia* H.W.Kung & G.L.Chu.

Annuals, shortly pubescent with simple hairs, branches not terminating in acicular apices. Leaves alternate, long-petiolate; leaf blade ovoid, entire, tip obtuse. Inflorescences leafy, axillary, glomerulate (clusters of 2 to 4 flowers). Flowers bisexual, supported by two bracteoles. Perianth green, of 5 almost free segments, keeled along midrib. Stamens 5, anthers small, without appendages. Stylodia 2. Fruit indehiscent, with foveolate surface after drying (mamillate when fresh), yellowish, crustaceous; pericarp tightly adjoining to the seed, mesocarp multicellular, composed of brachysclereids. Seeds yellowish, roundish, seed coat of two thin layers of equal thickness; embryo vertical.

A monotypic tribe consisting of *Baolia bracteata* H.W.Kung & G.L.Chu, a narrow endemic to Diebu [Têwo] county, Gansu province, China.

#### Note

*Baolia* seemed first to be related to *Chenopodium* [16], but later it was transferred to the Polycnemeae tribe (Amaranthaceae s.s.) with possible relations with *Polycnemum*, *Nitrophila* and *Hemichroa* [50]. Sukhorukov [18] proposed that *Baolia* is rather a member of Amaranthaceae s.s. or Caryophyllaceae based on the reproductive characters studied. In light of the recent molecular results, the morphoanatomical similarities are convergences in *Baolia*, some Caryophyllaceae and Amaranthaceae s.s.

## Materials and methods

### Taxon sampling, DNA extraction and sequencing of chloroplast genome

For *Baolia bracteata*, sixteen samples from two populations including seven and nine individuals, respectively, were used. These collections were made by SXG in 2021 in Diebu [Têwo] County, Gansu Province, China. Additionally, two *Corispermum* species, *C. chinganicum* and *C. declinatum*, were also sampled. No specific permissions were required for sampling and collection from these localities. Voucher specimens are deposited in the Herbarium of the Xinjiang Institute of Ecology and Geography Chinese Academy of Sciences (XJBI) and Tree Specimen Room of Forestry College, Gansu Agricultural University (GAUF) (Table S5 and Table S6). Plant identifications were conducted by SXG and WZB.

Young and fresh leaves were harvested and promptly preserved in silica gel. Genomic DNA was subsequently extracted from approximately 100 mg of silica-dried leaves following isolation protocols followed the modified 2 × CTAB buffer method [51]. The quality of the DNA was assessed using electrophoresis in a 1% (w/v) agarose gel. To construct a library, tags were assigned to each sample, and Illumina MiSeq / HiSeq2500 sequencing was employed [52]. The library’s fragment size ranged between 500 bp and 700 bp, with bidirectional 150–250 bp sequencing performed. Ensuring a minimum of 2 GB of sequencing data per species [53]. Moreover, the extracted DNA underwent sequencing using the ABI 3730xl DNA sequencer.

### Chloroplast genome assembly and annotation

GetOrganelle v1.7.5 was used with default parameters to assemble clean data [54]. Bandage v0.8.1 was utilized to confirm whether they were assembled into a ring structure [55]. The genomes of *Chenopodium acuminatum* Willd. (GenBank No. MW057780.1) and *Salsola collina* Pall. (GenBank No. OK189514.1) were selected as references. GeSeq v2.03 [56] and PGA (https://github.com/quxiaojian/PGA) [57] were employed for annotating the complete chloroplast genome and verifying sequencing accuracy. For sequence verification, BLAST v2.8.1 was employed [58].

Start and stop codons were manually adjusted, and pseudogenes were identified using Geneious v8.0.2 [59]. Genes with truncated, shortened, or deleted open reading frames, along with multiple stop codons, were classified as pseudogenes. The organelle genome drawing tool OGDRAW (http://ogdraw.mpimp-golm.mpg.de/) was used to create and visualize the circular plastid diagram [60, 61]. The accession numbers for the complete chloroplast genome sequences have been deposited in GenBank (Accession No. OR449093 - OR449108).

### Comparative analysis of chloroplast genomes

The software MAFFT v7 was utilized to compare the chloroplast genome [62]. The mVISTA program (http://genoes.lbl.gov/vista/mvista/submit.shtml) [63] was employed to assess differences in chloroplast genomes among various species, with *Baolia bracteata* serving as the reference. IRscope (https://irscope.shinyapps.io/irapp/) [64] was employed to compare chloroplast genome contractions and expansions between *B. bracteata* and other species. Rearrangements or inversions of fragments within the genome were identified using Mauve v2.4.0 with default settings [65]. Nucleotide polymorphism (Pi) values were evaluated using DnaSP v5 with window length set as the whole length of each matrix [66].

### Repetitive sequence analysis of chloroplast genomes

The REPuter program (https://bibiserv.cebitec.uni-bielefeld.de/reputer) [67] was employed to locate larger repetitive sequences, with the following parameters: Hamming distance of 3, a minimum repeat size of 30 bp, and a maximum computed repeat of 5,000 bp [68]. This search aimed to identify forward (F), reverse (R), palindromic (P), and complementary (C) repeats within the LSC, IRb, IRa, and SSC regions. For identifying SSRs in sixteen chloroplast genomes of *B. bracteata*, the misa tool (https://webblast.ipk-gatersleben.de/misa/index.php) [69] was employed, using the subsequent parameters: a minimum repeat threshold of 10 for mononucleotide (mono-) repeats, 5 for dinucleotide (di-) repeats, 4 for trinucleotide (tri-) repeats, and 3 for tetranucleotide (tetra-), pentanucleotide (penta-), and hexanucleotide (hexa-) repeat thresholds.

### Taxon sampling for targeted sanger sequencing

The nrITS region and two cp. markers, *rbcL* and *matK*, were employed in this study. Sequences from *B. bracteata* were extracted from each chloroplast genome using Geneious v8.0.2 [59] for the ITS sequence and both Geneious and PhyloSuite v1.2.2 [59, 70] for *rbcL* and *matK* sequences. Three representative samples from each population of *B. bracteata* were included. Ultimately, eighteen *B. bracteata* sequences were generated (Additional file 2: Table S6). A total of 236 published and new sequences, representing 91 species, were incorporated into the phylogenetic analyses. Among these, 80 species belong to Chenopodiaceae s.s., eight belong to Amaranthaceae, and three species were used as outgroups representing three different families: *Phaulothamnus spinescens* A.Gray (Achatocarpaceae), *Rhabdodendron amazonicum* (Spruce ex Benth.) Huber (Rhabdodendronaceae), and *Simmondsia chinensis* (Link) C.K.Schneid. (Simmondsiaceae).

### Phylogenetic analysis

For chloroplast genome data, we selected 33 chloroplast genomes from 18 species for analysis (Additional file 2: Table S5). We employed MAFFT v7 to compare all the complete chloroplast genomes, the gaps were deleted by Gblocks v.0.91b [68]. Subsequently, the best model GTRGAMMA was selected in jModelTest2 on XSEDE (2.1.6) with Bootstrap iterations set to 1,000 [71, 72]. Phylogenetic trees were constructed based on the maximum likelihood (ML) method in RAxML-HPC2 on XSEDE (8.2.12) [73]. To generate the Bayesian inference (BI) tree, we used MrBayes on XSEDE (3.2.7a) with the model TVM + I + G (lsetnst = 6 rates = invgamma) for selecting plastid intact sequences in the BI analyses [72–74]. We employed two independent Markov Chain Monte Carlo (MCMC) chains, running for 20 million generations with a sampling frequency of every 1,000 generations. The initial 25% of the sampled data was discarded for burn-in [75]. The constructed phylogenetic tree was visualized using FigTree v1.4.2 [76].

For gene fragment sequences data, sequences were aligned using MAFFT v7 and subsequently adjusted manually. Gaps were introduced into the alignment to represent missing data. Initially, we analyzed the nuclear (nrITS) and two plastid (*matK* and *rbcL*) datasets separately to detect any conflicts. Since no conflicts, we utilized the concatenated data of all three markers for this study. Phylogenetic analyses were conducting employing both the Maximum Likelihood (ML) and Bayesian Inference (BI) methods.

The ML support values were estimated through 1,000 bootstrap replicates. For the BI analysis, four chains were run (Markov Chain Monte Carlo), commencing with a random tree, and trees were saved every 100 generations for a total of 2 million generations. Prior to the ML and BI analyses, the appropriate model of DNA substitution was estimated using jModeltest v2.1 [73]. For the combined dataset, the TIM1 + I + G model was selected, with the gamma distribution shape parameter set to 0.6320. The base frequencies were specified as follows: A = 0.2811, C = 0.2060, G = 0.2241, and T = 0.2915. Both the ML and BI analyzes were conducting using the CIPRES Science Gateway v3.3 (https://www.phylo.org).

#### Divergence time estimation

Only species from the core Corispermoideae and Chenopodioideae as well as the genera *Acroglochin* and *Baolia* were included in the analyses. The sequences were aligned using MAFFT v7 and then manually adjusted. Gaps were introduced to the alignment as missing data. The two data sets, nuclear (nrITS) and plastid (*rbcL* + *matK*) were analyzed separately using BEAST v.1.8.2, respectively [77]. BEAUti was first used to set priors and created the BEAST.xml input files. For analyses, Chenopodioideae representatives were defined as monophyletic in order to set the root at the split between Chenopodioideae / (*Acroglochin* + *Baolia* + Corispermeae). The substitution model parameters were set to HKY + I + G for *rbcL* + *matK* dataset, GTR + G for nrITS dataset based on the program jModelTest2. The relaxed Bayesian clock was implemented with rates for each branch drawn independently from a lognormal distribution [78]. A birth and death prior was set for branch lengths. The root age was set to 57 − 55 mya [1, 6] using the normal prior. Due to the differences between the previous estimation of crown age of Atripliceae [s.str.] based on *rbcL* + *matK* dataset and ITS dataset, the crown age of Atripliceae was set to 31-16.4 mya, 29.4–19.2 mya in *rbcL* + *matK* dataset, ITS dataset, respectively [79]. The first runs were used to examine MCMC performance, and operators were adjusted as suggested by the output analysis. The final run was performed with 50,000,000 interations for ITS dataset, and 100,000,000 interations for *rbcL* + *matK* dataset, a burn-in of 10% and a sample frequency of 1,000. The Bayes factor was calculated by Tracer v1.7.2 [80] to check the effective sample sizes (> 200), and then the maximum clade credibility tree was generated in TreeAnnotator v1.8.2 [77] with a posterior probability limit of 0.7 and generated mean node heights. Final trees were edited in Figtree v1.4.2.

### Ancestral character reconstruction

Ancestral characters of *Baolia* and related genera were reconstructed based on the pruned maximum clade credibility Bayesian tree generated above. Taxa with more than 70% missing data in the character matrix and duplicate samples were pruned using the drop.tip function in R [81]. The character matrix included ten coded discrete characters that are significant in the taxonomy of Amaranthaceae (Additional file 2: Table S7). Ancestral state reconstructions were carried out using the MrBayes Ancestral States with R [82]. Similar to the native MrBayes, MBASR employs continuous-time Markov modeling against a tree’s topology and branch lengths to statistically estimate for character states at ancestral tree nodes for discrete traits [82]. All analyses were performed in R v.4.2.2.

#### Morphoanatomical studies

The morphoanatomical data for *Agriophyllum*, *Anthochlamys* and *Corispermum* (Corispermoideae) were obtained from previous detailed studies [44, 83]. Carpological features of *Baolia* and *Acroglochin* were examined by preparing cross-sections using a Microm HM 355 S rotary microtome (Thermo Fisher Scientific, USA). Prior to sectioning, the material was immersed in water: alcohol: glycerin (1: 1: 1) solution, dehydrated in a series of ethanol dilutions and embedded in Technovit 7100 resin (Heraeus Kulzer, Germany). The cross-sections were examined using a Nikon Eclipse Ci microscope and captured with a Nikon DS-Vi1 camera (Nikon Corporation, Japan). The fruit and seed surface was examined using a scanning electron microscope (SEM) JSM-6380 (JEOL Ltd., Japan) at 15 kV after sputter-coating with gold-palladium using an EIKO IB-3 Ion Coater (EIKO Engineering Ltd., Japan) at the Electron Microscopy laboratory, M.V. Lomonosov Moscow State University. Before SEM imaging, *Baolia* fruit underwent dehydration in aqueous ethyl alcohol solutions of increasing concentrations, followed by alcohol-acetone solutions, and pure acetone. Ten carpological characters and their states were coded in the present study for *Acroglochin*, *Baolia*, three Corispermeae (*Corispermum*, *Anthochlamys*, and *Agriophyllum*) and Chenopodioideae (see Additional file 2: Table S7).

#### Distribution mapping

Herbarium specimens of *Acroglochin* and *Baolia* stored at B, BM, BR, BSD, CAH, CDBI, DD, E, FJS, G, H, HUJ, IBSC, IMC, JIU, K, KATH, L (including U and WAG), KUN, LE, LY, M, MHA, MSB, MW, NAS, P, PE, PRA, SHI, TO, TUCH, W, WU, WUK, XIA, and XJBI were analyzed (herbarium abbreviations according to Thiers 2023+). The herbarium specimens of *Acroglochin* collected by APS are located in MW. Distribution maps are based on the specimens seen, and these were prepared using SimpleMappr online tool (http://www.simplemappr.net).

### Supplementary Information

Supplementary Material 1.

Supplementary Material 2.

## Data Availability

All plastomes generated in this study are deposited in NCBI database (https://www.ncbi.nlm.nih.gov/) (GenBank accession Nos. OP584480-OP584485, OP584905-OP584916, OR449093-OR449108, OR458831-OR458832, see Table S5 and Table S6). These data will remain private until the related manuscript has been accepted.

## Abbreviations

BI: Bayesian inference
bp: Base pair
bs: Bootstrap support
cp: Chloroplast
CTAB: Cetyl trimethylammonium bromide
GC: Guanine-cytosine
GTR: General time reversible
IGS: Intergenic regions
ITS: Internal transcribed spacer of ribosomal DNA
IR: Inverted repeat
IRs: Inverted repeat regions
IRa, IRb: Two IR regions that are identical but in opposite orientations
kb: Kilobase
LSC: Large single copy
MCMC: Markov chain Monte Carlo
ML: Maximum Likelihood
NCBI: National Center for Biotechnology Information
PGA: Plastid Genome Annotator
pP: Posterior probability
rRNA: Ribosomal RNA
SSC: Small single copy
SSRs: Simple sequence repeat
tRNA: Transfer RNA
