# Supplementary material for: Phylogenetic placement of the monotypic Baolia (Amaranthaceae s.l.) based on morphological and molecular evidence
Source: BMC Plant Biol. 2024 May 25;24:456. doi: 10.1186/s12870-024-05164-8 (PMC11127444; doi:10.1186/s12870-024-05164-8)
Supplement: Supplementary file 1 — Supplementary Material 1. [file 12870_2024_5164_MOESM1_ESM.zip › Fig. S14_ Ancestral character reconstruction of Sclerenchymatous tissue in the pericarp characters in Corispermoideae.pdf]

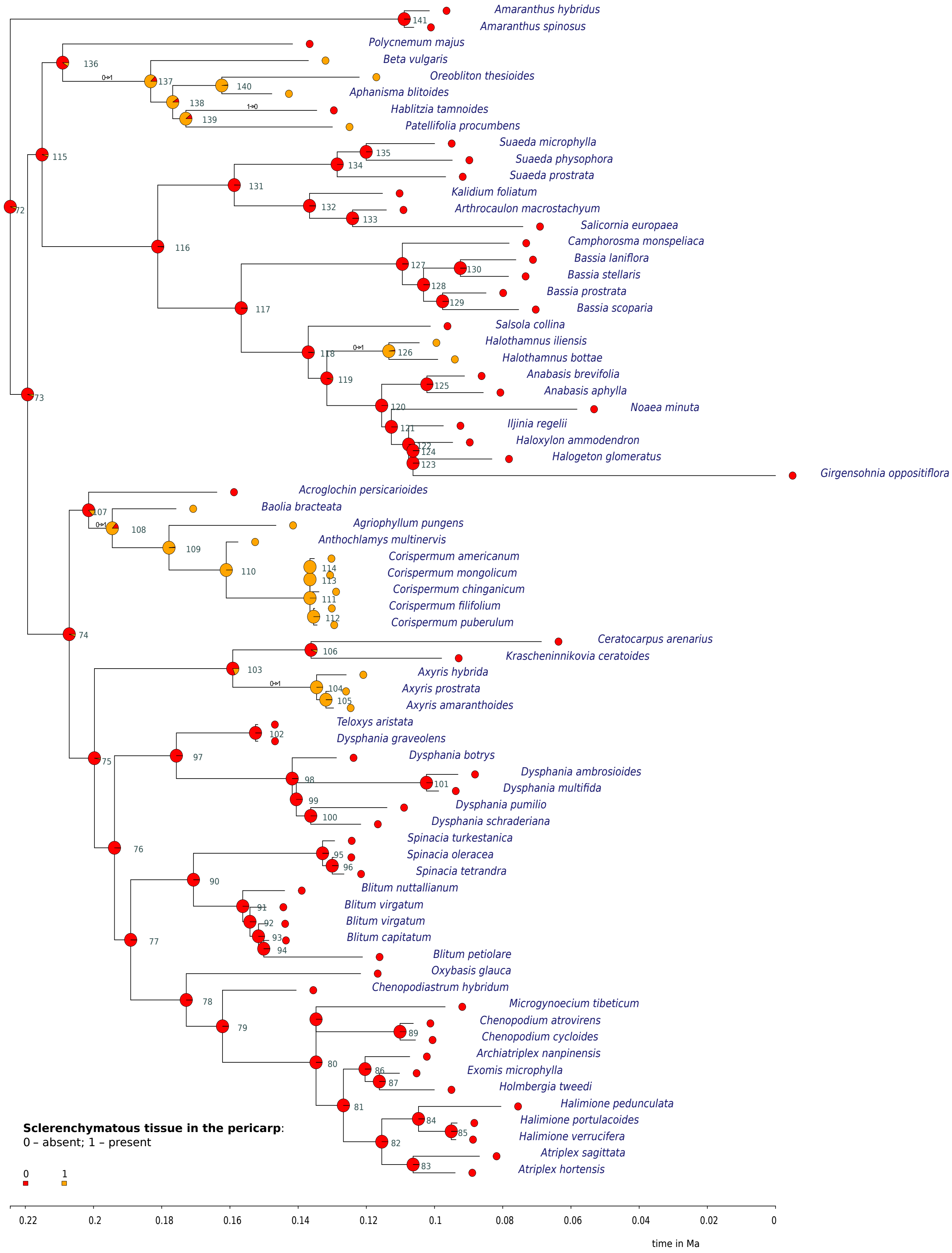


Ancestral character reconstruction of pericarp with sclerenchymatous tissue in Corispermoideae. Number on the nodes correspond to the node numbers while numbers above the nodes with arrows indicate transition of states.
