# Supplementary material for: Phylogenetic placement of the monotypic Baolia (Amaranthaceae s.l.) based on morphological and molecular evidence
Source: BMC Plant Biol. 2024 May 25;24:456. doi: 10.1186/s12870-024-05164-8 (PMC11127444; doi:10.1186/s12870-024-05164-8)
Supplement: Supplementary file 1 — Supplementary Material 1. [file 12870_2024_5164_MOESM1_ESM.zip › Fig. S4_Phylogenetic tree reconstruction of the 97 species inferred from maximum likelihood and Bayesian analyses based on the three molecular markers (ITS, rbcL, and matK).pdf]

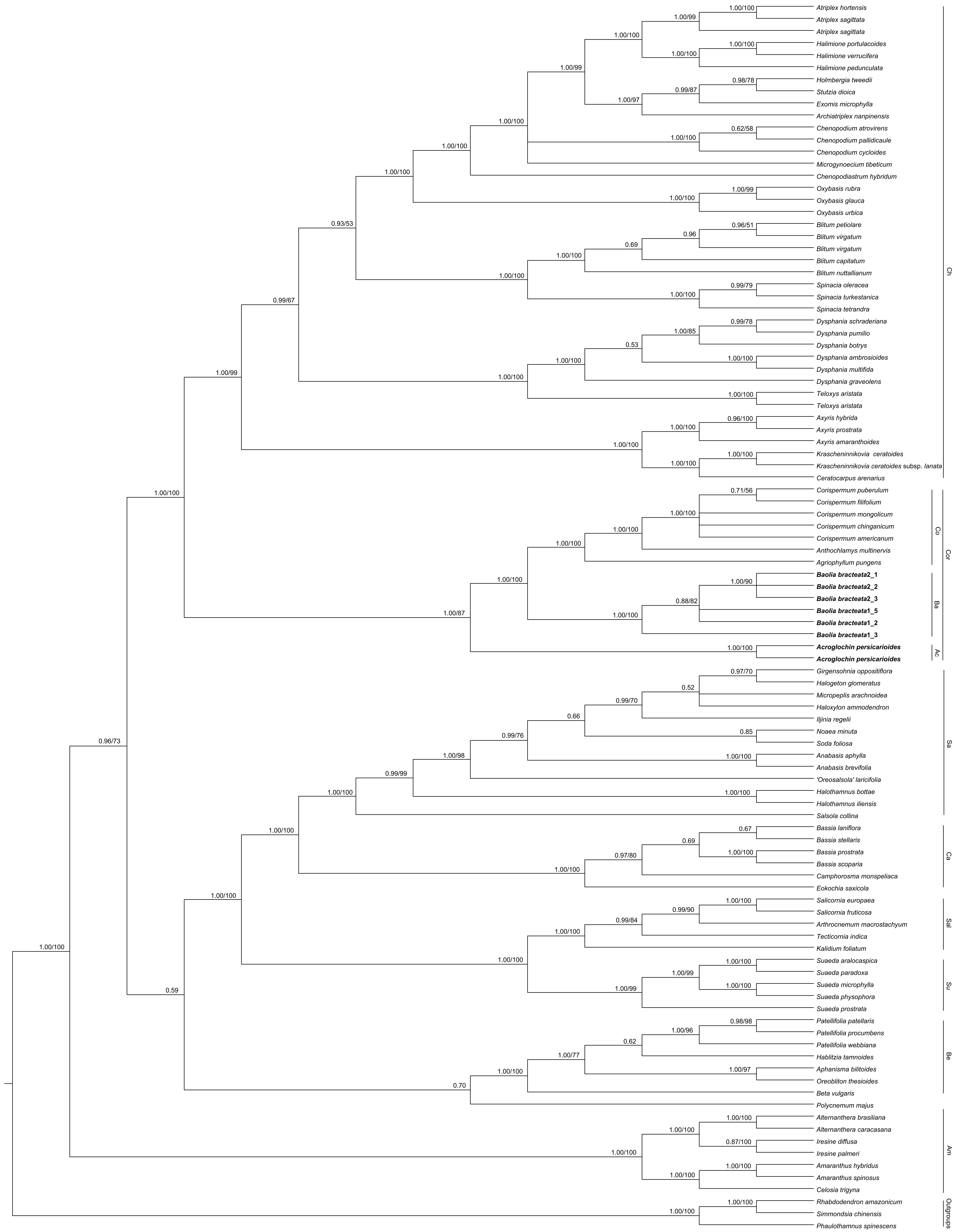

Phylogenetic tree reconstruction of the 97 species inferred from maximum likelihood and Bayesian analyses based on the three molecular markers (ITS, *rbc*L, and *mat*K). Bayesian posterior probabilities / ML bootstrap values are shown above branches. Only the values greater 0.5 or 50% appear in the tree. Abbreviation: Ac, Acroglochineae; Am, Amaranthaceae s.s.; Ba, Baolieae; Be, Betoideae; Ca, Camphorosmoideae; Co, Corispermeae; Cor, Corispermoideae; Ch, Chenopodioide-ae; Sa, Salsoloideae; Sal, Salicornioideae; Su, Suaedoideae.
