# Supplementary material for: Phylogenetic placement of the monotypic Baolia (Amaranthaceae s.l.) based on morphological and molecular evidence
Source: BMC Plant Biol. 2024 May 25;24:456. doi: 10.1186/s12870-024-05164-8 (PMC11127444; doi:10.1186/s12870-024-05164-8)
Supplement: Supplementary file 1 — Supplementary Material 1. [file 12870_2024_5164_MOESM1_ESM.zip › Fig. S6_Phylogeny of Baolieae, Corispermeae and Acroglochineae based on ITS sequence data and a BEAST analysis.pdf]

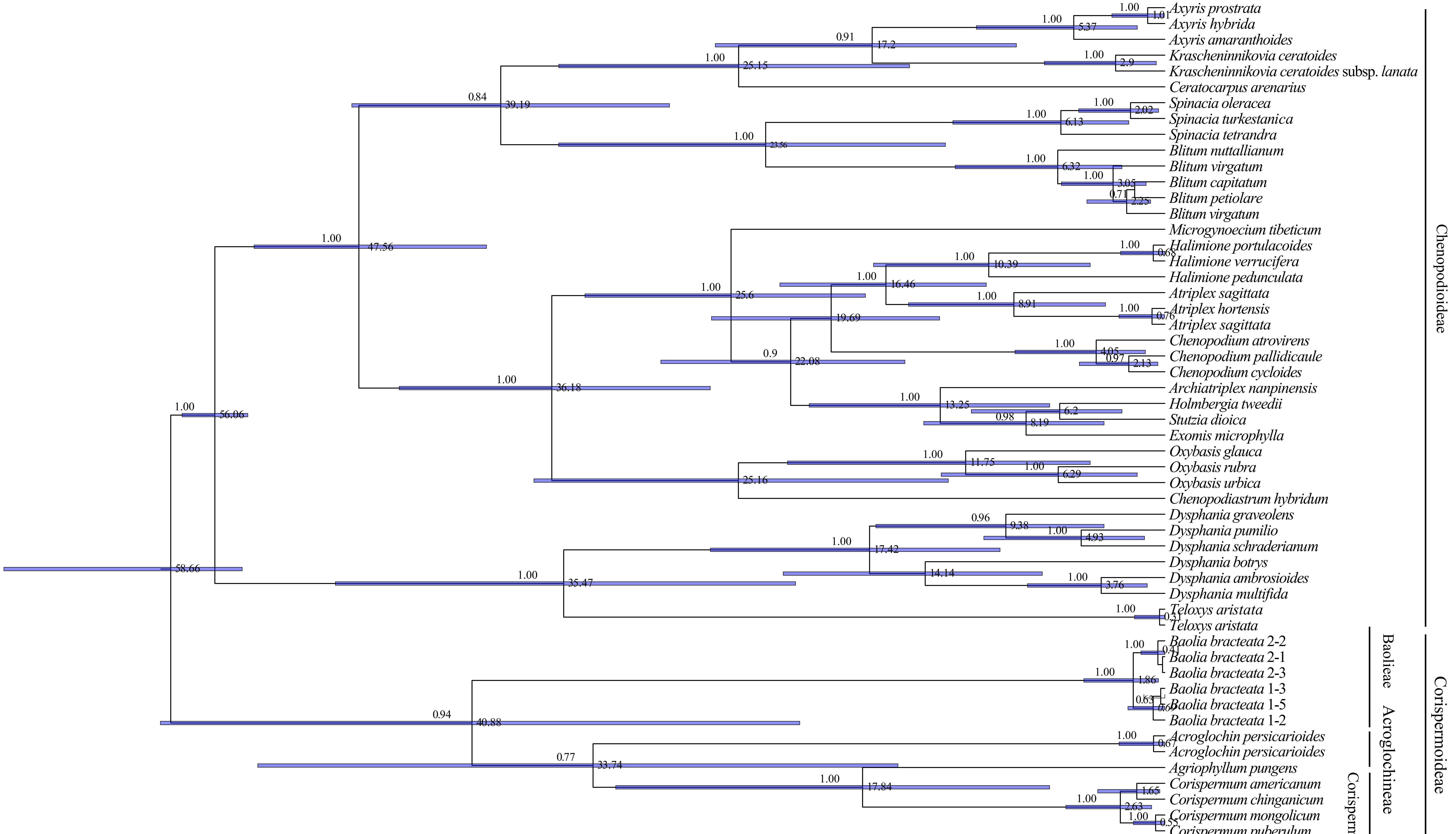

Phylogeny of Baolieae, Corispermeae and Acroglochineae based on ITS sequence data and a BEAST analysis. Posterior probabilities higher than 0.7 are appeared above branches, node ages estimated are given behind the respective node, and the corresponding bars represent 95% confidence intervals.
