# Supplementary figures and images for: Phylogenetic placement of the monotypic Baolia (Amaranthaceae s.l.) based on morphological and molecular evidence
Source: BMC Plant Biol. 2024 May 25;24:456. doi: 10.1186/s12870-024-05164-8 (PMC11127444; doi:10.1186/s12870-024-05164-8)

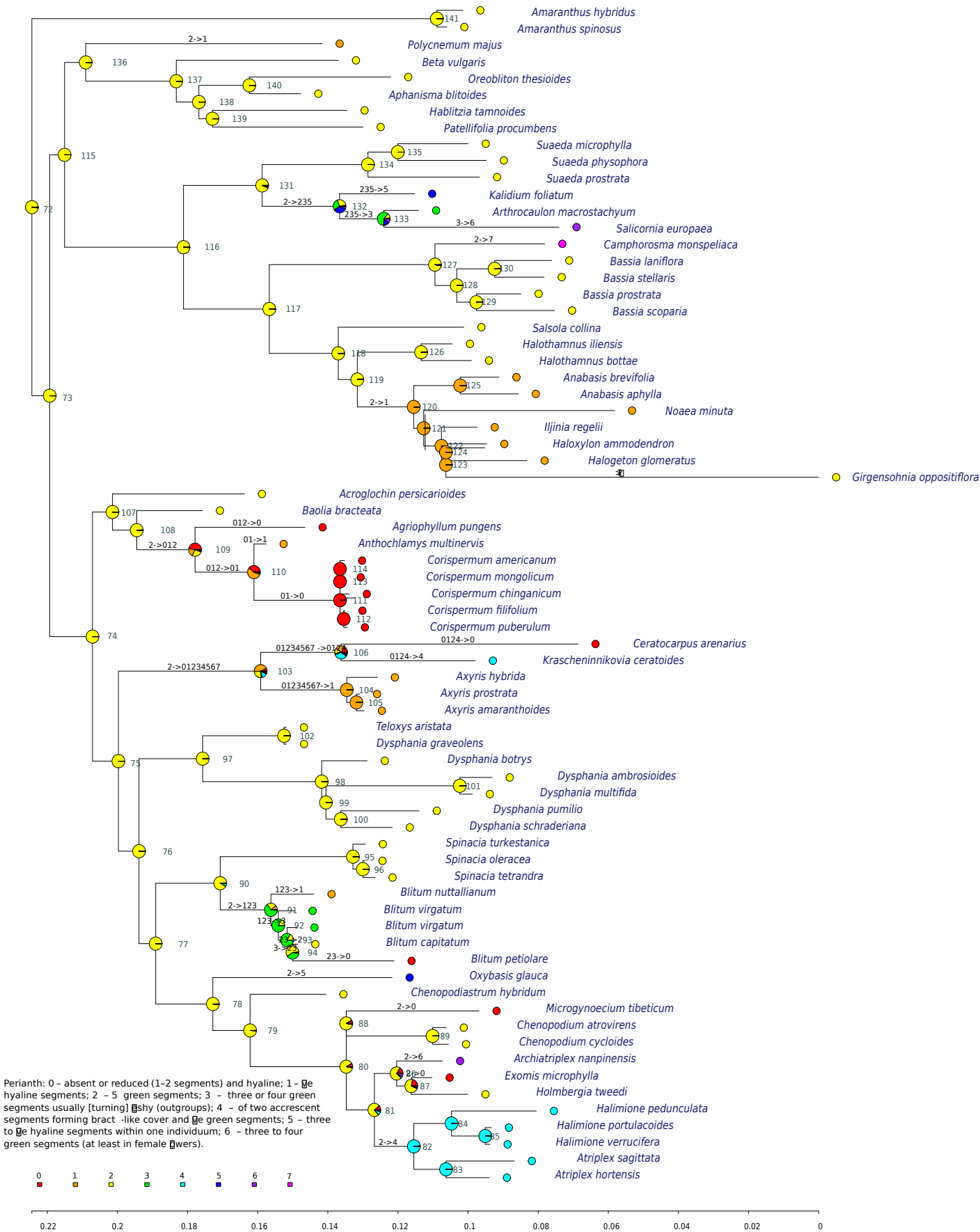

Supplement: Supplementary file 1 — Supplementary Material 1. [file 12870_2024_5164_MOESM1_ESM.zip › Fig. S11_Ancestral character reconstruction of perianth characters in Corispermoideae.pdf]

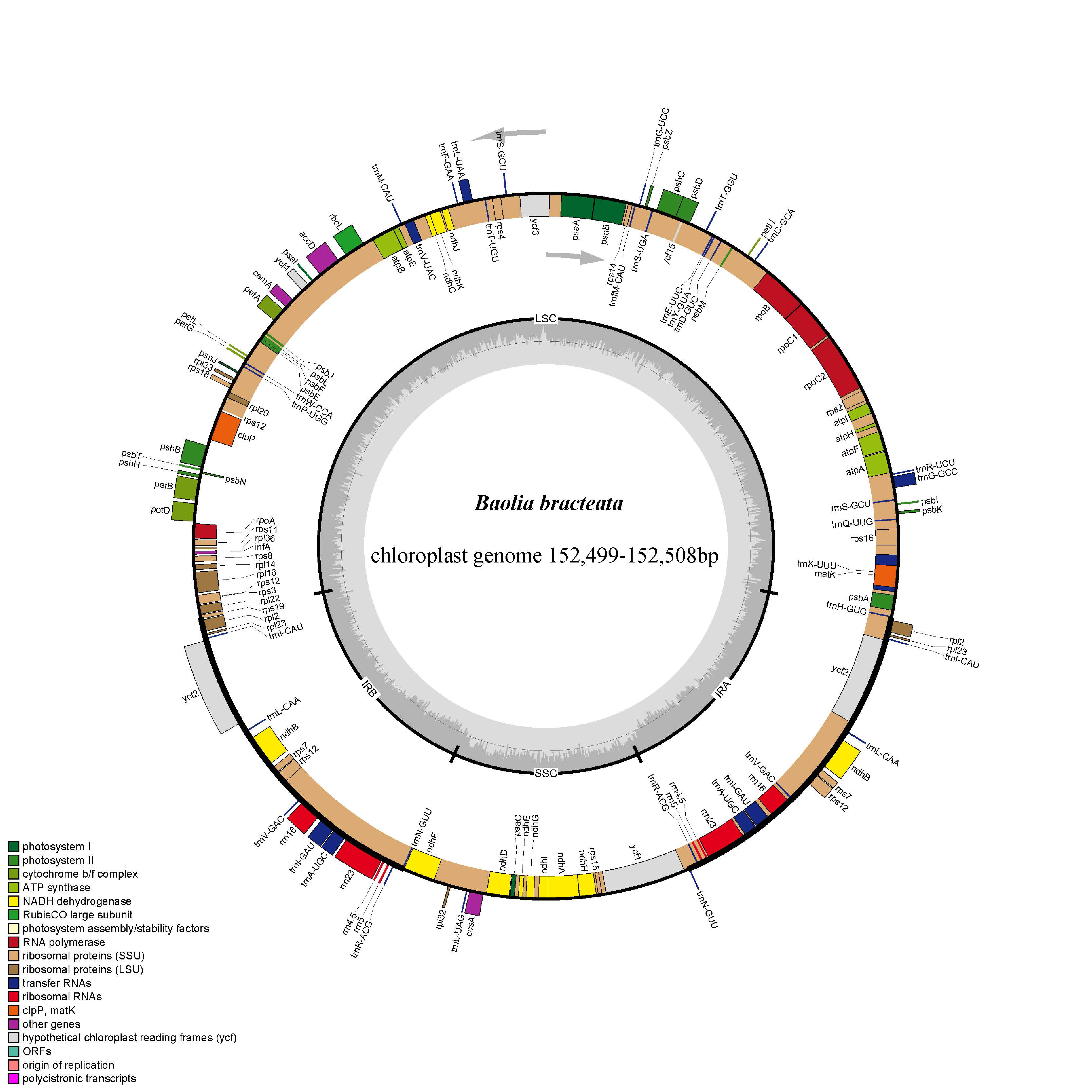

Supplement: Supplementary file 1 — Supplementary Material 1. [file 12870_2024_5164_MOESM1_ESM.zip › Fig. S1_gene map of the Baolia bracteata chloroplast genomes.jpg]

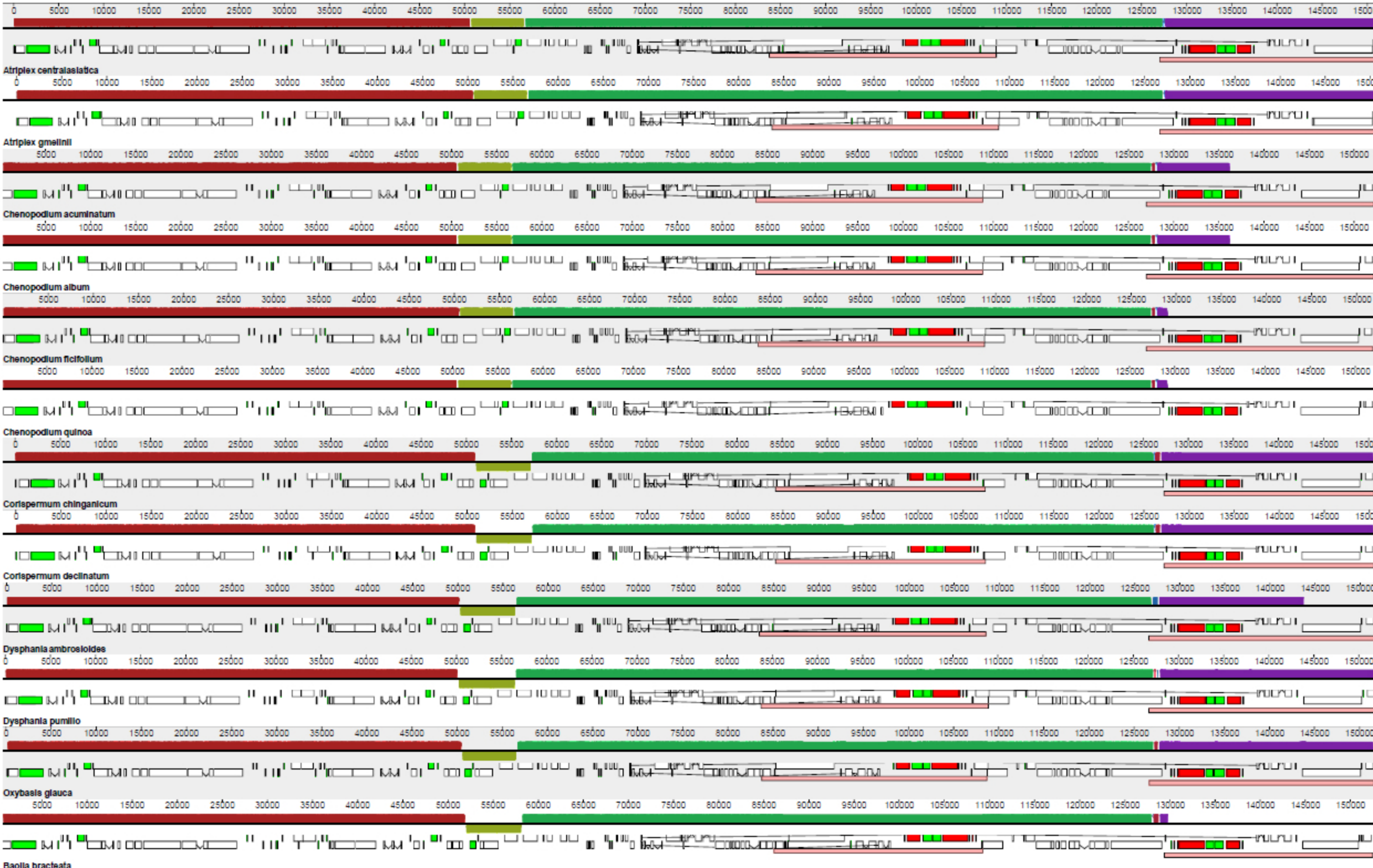

Supplement: Supplementary file 1 — Supplementary Material 1. [file 12870_2024_5164_MOESM1_ESM.zip › Fig. S3_Use the Mauve multi genome alignment program to detect the collinearity and chloroplast genome structure of 12 species genomes.pdf]
