# Supplementary material for: Phylogenetic placement of the monotypic Baolia (Amaranthaceae s.l.) based on morphological and molecular evidence
Source: BMC Plant Biol. 2024 May 25;24:456. doi: 10.1186/s12870-024-05164-8 (PMC11127444; doi:10.1186/s12870-024-05164-8)
Supplement: Supplementary file 2 — Supplementary Material 2. [file 12870_2024_5164_MOESM2_ESM.zip › Table S2_List of genes in the chloroplast genomes of the Baolia bracteata species.docx]

Table S2 List of genes in the chloroplast genomes of the *Baolia bracteata* species

| Category | Gene group | Gene name |
| --- | --- | --- |
| Photosynthesis | Subunits of photosystem I | psaA, psaB, psaC, psaI, psaJ |
|  | Subunits of photosystem II | psbA, psbB, psbC, psbD, psbE, psbF, psbH, psbI, psbJ, psbK, psbL, psbM, psbN, psbT, psbZ |
|  | Subunits of NADH dehydrogenase | ndhA*, ndhB*(2), ndhC, ndhD, ndhE, ndhF, ndhG, ndhH, ndhI, ndhJ, ndhK |
|  | Subunits of cytochrome b/f complex | petA, petB*, petD*, petG, petL, petN |
|  | Subunits of ATP synthase | atpA, atpB, atpE, atpF*, atpH, atpI |
|  | Large subunit of rubisco | rbcL |
|  | Subunits photochlorophyllide reductase | - |
| Self-replication | Proteins of large ribosomal subunit | rpl14, rpl16*, rpl2(2), rpl20, rpl22, #rpl23(2), rpl32, rpl33, rpl36 |
|  | Proteins of small ribosomal subunit | rps11, rps12**(2), rps14, rps15, rps16*, rps18, #rps19, rps2, rps3, rps4, rps7(2), rps8 |
|  | Subunits of RNA polymerase | rpoA, rpoB, rpoC1*, rpoC2 |
|  | Ribosomal RNAs | rrn16(2), rrn23(2), rrn4.5(2), rrn5(2) |
|  | Transfer RNAs | trnA-UGC*(2), trnC-GCA, trnD-GUC, trnE-UUC, trnF-GAA, trnG-GCC*, trnG-UCC, trnH-GUG, trnI-CAU(2), trnI-GAU*(2), trnK-UUU*, trnL-CAA(2), trnL-UAA*, trnL-UAG, trnM-CAU, trnN-GUU(2), trnP-UGG, trnQ-UUG, trnR-ACG(2), trnR-UCU, trnS-GCU(2), trnS-UGA, trnT-GGU, trnT-UGU, trnV-GAC(2), trnV-UAC*, trnW-CCA, trnY-GUA, trnfM-CAU |
| Other genes | Maturase | matK |
|  | Protease | clpP** |
|  | Envelope membrane protein | cemA |
|  | Acetyl-CoA carboxylase | accD |
|  | c-type cytochrome synthesis gene | ccsA |
|  | Translation initiation factor | infA |
| Genes of unknown function | Conserved hypothetical chloroplast ORF | ycf1, ycf15, ycf2(2), ycf3**, ycf4 |

| Note: Gene*, Gene with one introns; Gene**, Gene with two introns; #Gene, Pseudo gene; Gene(2): Number of copies of multi-copy genes. |
| --- |
