# Supplementary material for: Phylogenetic placement of the monotypic Baolia (Amaranthaceae s.l.) based on morphological and molecular evidence
Source: BMC Plant Biol. 2024 May 25;24:456. doi: 10.1186/s12870-024-05164-8 (PMC11127444; doi:10.1186/s12870-024-05164-8)
Supplement: Supplementary file 2 — Supplementary Material 2. [file 12870_2024_5164_MOESM2_ESM.zip › Table S5_Species used in this study including phylogeny and comparative chloroplast genome analysis.docx]

Table S5. Species used in this study including phylogeny and comparative chloroplast genome analysis

| **Species** | **Species ID** | **Herbarium/Voucher No.** | **Localities** | **Locations** |  | **NCBI number** |
| --- | --- | --- | --- | --- | --- | --- |
|  |  |  |  | **N** | **E** |  |
| *Baolia bracteata* H.W.Kung & G.L.Chu | 1-1 | GAUF/742 | China: Gansu | 33.946 | 103.736 | OR449093 |
| *Baolia bracteata* H.W.Kung & G.L.Chu | 1-2 | GAUF/742 | China: Gansu | 33.946 | 103.736 | OR449094 |
| *Baolia bracteata* H.W.Kung & G.L.Chu | 1-3 | GAUF/742 | China: Gansu | 33.946 | 103.736 | OR449095 |
| *Baolia bracteata* H.W.Kung & G L.Chu | 1-4 | GAUF/742 | China: Gansu | 33.946 | 103.736 | OR449096 |
| *Baolia bracteata* H.W.Kung & G.L.Chu | 1-5 | GAUF/742 | China: Gansu | 33.946 | 103.736 | OR449097 |
| *Baolia bracteata* H.W.Kung & G.L.Chu | 1-6 | GAUF/742 | China: Gansu | 33.946 | 103.736 | OR449098 |
| *Baolia bracteata* H.W.Kung & G.L.Chu | 1-7 | GAUF/742 | China: Gansu | 33.946 | 103.736 | OR449099 |
| *Baolia bracteata* H.W.Kung & G.L.Chu | 1-8 | GAUF/742 | China: Gansu | 33.946 | 103.736 | OR449100 |
| *Baolia bracteata* H.W.Kung & G.L.Chu | 1-9 | GAUF/742 | China: Gansu | 33.946 | 103.736 | OR449101 |
| *Baolia bracteata* H.W.Kung & G.L.Chu | 2-1 | GAUF/743 | China: Gansu | 34.004 | 103.933 | OR449102 |
| *Baolia bracteata* H.W.Kung & G.L.Chu | 2-2 | GAUF/743 | China: Gansu | 34.004 | 103.933 | OR449103 |
| *Baolia bracteata* H.W. Kung & G.L.Chu | 2-3 | GAUF/743 | China: Gansu | 34.004 | 103.933 | OR449104 |
| *Baolia bracteata* H.W. Kung & G.L.Chu | 2-4 | GAUF/743 | China: Gansu | 34.004 | 103.933 | OR449105 |
| *Baolia bracteata* H.W.Kung & G.L.Chu | 2-5 | GAUF/743 | China: Gansu | 34.004 | 103.933 | OR449106 |
| *Baolia bracteata* H. W. Kung & G.L.Chu | 2-6 | GAUF/743 | China: Gansu | 34.004 | 103.933 | OR449107 |
| *Baolia bracteata* H.W.Kung & G.L.Chu | 2-7 | GAUF/743 | China: Gansu | 34.004 | 103.933 | OR449108 |
| *Corispermum chinganicum* Iljin | 103 | XJBI/wzb-103 | China: Ningxia | 38.603 | 105.952 | OR458831 |
| *Corispermum declinatum* Stephan ex Iljin | 104 | XJBI/wzb-124 | China: Xinjiang | 44.044 | 93.082 | OR458832 |
| *Chenopodium acuminatum* Willd. | Wariss and Qu (2021) | | | | | MW057780 |
| *Chenopodium album* L. | Park et al. (2021) | | | | | MW446246 |
| *Chenopodium ficifolium* Sm. | Kim and Park (direct submission to Genbank) | | | | | MK182725 |
| *Chenopodium quinoa* Willd. | Gao et al. (2021) | | | | | MT906655 |
| *Atriplex centralasiatica* Iljin | Zhang et al (2019) | | | | | MK867774 |
| *Atriplex gmelinii* C.A.Mey. | Park et al. (2022) | | | | | MT810472 |
| *Dysphania ambrosioides* (L.) Moskin & Clemants | Kim and Park (direct submission to Genbank) | | | | | NC041201 |
| *Dysphania pumilio* (R.Br.) Moskin & Clemants | Park et al. (2021) | | | | | MK541016 |
| *Oxybasis glauca* (L.) Fuentes, Uotila & Borsch | Kim et al. (direct submission to Genbank) | | | | | NC047226 |
| *Kalidum foliatum* (Pall.) Moq. | Zhang (direct submission to Genbank) | | | | | OL342319 |
| *Salicornia bigelovii* Torr. | Jamdade et al. (2022) | | | | | KJ629117 |
| *Suaeda glauca* Bunge | Qu et al. (2019) | | | | | MK867773 |
| *Caroxylon passerinum* (Bunge) Akhani & Roalson | Xie et al. (2022) | | | | | MW192441 |
| *Salsola collina* Pall. | Liu et al. (2023) | | | | | OK189514 |
| *Haloxylon persicum* Bunge | Suo (direct submission to Genbank) | | | | | KF534479 |
