# Supplementary material for: Phylogenetic placement of the monotypic Baolia (Amaranthaceae s.l.) based on morphological and molecular evidence
Source: BMC Plant Biol. 2024 May 25;24:456. doi: 10.1186/s12870-024-05164-8 (PMC11127444; doi:10.1186/s12870-024-05164-8)
Supplement: Supplementary file 2 — Supplementary Material 2. [file 12870_2024_5164_MOESM2_ESM.zip › Table S6_Species used in phylogeny study based on the gene fragment sequences.docx]

Table S6 Species used in phylogeny study based on the gene fragment sequences

| **Species** | **Species ID** | **Herbarium/Voucher No.** | **Localities** | **Locations** |  | **ITS NCBI number** | ***rbc*L NCBI number** | *mat*K NCBI number |
| --- | --- | --- | --- | --- | --- | --- | --- | --- |
|  |  |  |  | **N** | **E** |  |  |  |
| **Chenopodiaceae s.s.** |  |  |  |  |  |  |  |  |
| **Acroglochinoideae** |  |  |  |  |  |  |  |  |
| *Acroglochin persicarioides* (Poir.) Moq. | Kadereit et al. (2006); Kadereit et al. (2003); Muller and Borsch (direct submission to Genbank) | | | | | AY858589 | AY270049 | AY514826 |
| *Acroglochin persicarioides* (Poir.) Moq. | Xu et al. (direct submission to Genbank) | | | | | MH808276 | MN192573 | MK435782 |
| **Betoideae** |  |  |  |  |  |  |  |  |
| *Aphanisma bilitoides* Nutt. ex Moq. | Hohmann et al. (2006); Kadereit et al. (2003); Muller and Borsch (direct submission to Genbank) | | | | | AY858591 | AY270057 | AY514844 |
| *Beta vulgaris* L. | Hohmann et al. (2006); Kadereit et al. (2003); Muller and Borsch (direct submission to Genbank) | | | | | AY858597 | AY270065 | AY514832 |
| *Hablitzia tamnoides* M. Bieb. | Hohmann et al. (2006); Kadereit et al. (2003); Muller and Borsch (direct submission to Genbank) | | | | | AY858590 | AY270092 | AY514825 |
| *Oreobliton thesioides* Durieu & Moq. | Hohmann et al. (2006); Kadereit et al. (2003) | | | | | AY858592 | AY270113 | ** |
| *Patellifolia patellaris* (Moq.) A. J. Scott | Hohmann et al. (2006); Kadereit and Freitag (2011); Romeiras et al. (2016) | | | | | AY858593 | HM630103 | LK054319 |
| *Patellifolia procumbens* (Chr. Sm.) A. J. Scott | Hohmann et al. (2006); Vieira (direct submission to Genbank); Romeiras et al. (2016) | | | | | AY858594 | LK054392 | LK054318 |
| *Patellifolia webbiana* (Moq.) A. J. Scott | Fujito et al. (2015); Romeiras et al. (2016) | | | | | AB935689 | LK054386 | LK054317 |
| **Camphorosmoideae** |  |  |  |  |  |  |  |  |
| *Bassia laniflora* (S. G. Gmel.) A. J. Scot | Kadereit et al. (2014) | | | | | KF785942 | ** | ** |
| *Bassia prostrata* (L.) Beck | Li (direct submission to Genbank); Kadereit et al. (2003) | | | | | MW725183 | AY270104 | ** |
| *Bassia scoparia* (L.) A. J. Scott | Li (direct submission to Genbank); Su and Cheng (2018); Christin (2011) | | | | | MW725162 | MH445312 | FR775278 |
| *Bassia stellaris* (Moq.) Bornm. | Kadereit et al. (2005) | | | | | AY489219 | ** | ** |
| *Camphorosma monspeliaca* L. | Roalson et al. (2007); Kadereit et al. (2003); Mueller and Borsch (direct submission to Genbank) | | | | | EF453392 | AY270071 | AY514829 |
| *Eokochia saxicola* Freitag & G. Kadereit | Kadereit et al. (2005); Kadereit and Freitag (2011) | | | | | AY489217 | HM630100 | ** |
| **Corispermoideae** |  |  |  |  |  |  |  |  |
| **Baolieae** |  |  |  |  |  |  |  |  |
| *Baolia bracteata* H. W. Kung & G. L. Chu | 1-2 | GAUF/742 | China: Gansu | 33.946 | 103.736 | OP584483 | OP584905 | OP584911 |
| *Baolia bracteata* H. W. Kung & G. L. Chu | 1-3 | GAUF/742 | China: Gansu | 33.946 | 103.736 | OP584484 | OP584906 | OP584912 |
| *Baolia bracteata* H. W. Kung & G. L. Chu | 1-5 | GAUF/742 | China: Gansu | 33.946 | 103.736 | OP584485 | OP584916 | OP584910 |
| *Baolia bracteata* H. W. Kung & G. L. Chu | 2-1 | GAUF/743 | China: Gansu | 34.004 | 103.933 | OP584480 | OP584907 | OP584913 |
| *Baolia bracteata* H. W. Kung & G. L. Chu | 2-2 | GAUF/743 | China: Gansu | 34.004 | 103.933 | OP584481 | OP584908 | OP584914 |
| *Baolia bracteata* H. W. Kung & G. L. Chu | 2-3 | GAUF/743 | China: Gansu | 34.004 | 103.933 | OP584482 | OP584909 | OP584915 |
| **Corispermeae** |  |  |  |  |  |  |  |  |
| *Agriophyllum pungens* (L.) Moq. | Genievskaya et al. (2017); Kadereit et al. (2003); Muller and Borsch (direct submission to Genbank) | | | | | MG282027 | AY270051 | AY514827 |
| *Anthochlamys multinervis* Rech. f. | Kadereit et al. (2003) | | | | | ** | AY270056 | ** |
| *Corispermum mongolicum* Iljin | Xue and Zhang (2011) | | | | | JF792734 | JF792783 | ** |
| *Corispermum chinganicum* Iljin | Xue and Zhang (2011) | | | | | JF792743 | JF792793 | ** |
| *Corispermum americanum* (Nutt.) Nutt. | Thornhill et al. (direct submission to Genbank) | | | | | MF964002 | MF963261 | MF963623 |
| *Corispermum puberulum* Iljin | Xue and Zhang (2011); Yao et al. (2017) | | | | | JF792749 | JF792799 | MF063954 |
| *Corispermum filifolium* C. A. Mey. | Kadereit et al. (2003) | | | | | ** | AY270084 | ** |
| **Chenopodioideae** |  |  |  |  |  |  |  |  |
| *Archiatriplex nanpinensis* G. L. Chu | Kadereit et al. (2010) | | | | | HM587476 | HM587580 | ** |
| *Atriplex hortensis* L. | Kadereit et al. (2010); Christin (2011) | | | | | HM005855 | FR775290 | FR775272 |
| *Atriplex sagittata* Borkh. | Fuentes-Bazan et al. (2012) | | | | | HE577361 | ** | ** |
| *Atriplex sagittata* Borkh. | Kadereit et al. (2010); Fuentes-Bazan et al. (2012) | | | | | HM587550 | ** | HE855637 |
| *Axyris amaranthoides* L. | Sukhorukov et al. (2022); Bennett et al. (2013); Fenneman and Graham (direct submission to Genbank) | | | | | ON775478 | JX848450 | KX677044 |
| *Axyris hybrida* L. | Sukhorukov et al. (2022); Cuenoud et al. (2002) | | | | | ON775485 | ** | AY042551 |
| *Axyris prostrata* L. | Sukhorukov et al. (2022); Kadereit et al. (2003); Fuentes-Bazan et al. (2012) | | | | | ON775489 | AY270062 | HE855600 |
| *Blitum capitatum* L. | Fuentes-Bazan et al. (2012); Kuzmina et al. (2012); Fuentes-Bazan et al. (direct submission to Genbank) | | | | | HE577373 | JN965445 | HE855614 |
| *Blitum nuttallianum* Schult. | Fuentes-Bazan et al. (2012); Kadereit et al. (2003); Fuentes-Bazan et al. (direct submission to Genbank) | | | | | HE577375 | AY270108 | HE855621 |
| *Blitum petiolare* Link | Sukhorukov et al. (2018); Orr et al. (direct submission to Genbank) | | | | | MH150883 | LT576806 | ** |
| *Blitum virgatum* L. | Kolano et al. (direct submission to Genbank); Kuzmina et al. (2017); Fuentes-Bazan et al. (direct submission to Genbank) | | | | | KJ629063 | MG246370 | ** |
| *Blitum virgatum* L. | Li et al. (2011); Kadereit et al. (2003); Fuentes-Bazan et al. (direct submission to Genbank) | | | | | JF976148 | AY270081 | HE855618 |
| *Ceratocarpus arenarius* L. | Zhou et al. (2007); Kadereit et al. (2010); Fuentes-Bazan et al. (direct submission to Genbank) | | | | | AY556430 | HM587594 | HE855601 |
| *Chenopodiastrum hybridum* (L.) S. Fuentes, Uotila & Borsch | Li (direct submission to Genbank); de Vere et al. (direct submission to Genbank); Fuentes-Bazan et al. (direct submission to Genbank) | | | | | MW725211 | MK924837 | HE855635 |
| *Chenopodium atrovirens* Rydb. | Sukhorukov et al. (2018); Fuentes-Bazan et al. (direct submission to Genbank) | | | | | KP226648 | KX679232 | HE855642 |
| *Chenopodium cycloides* A. Nelson | Fuentes-Bazan et al. (2012); Fuentes-Bazan et al. (direct submission to Genbank) | | | | | HE577460 | ** | HE855643 |
| *Chenopodium pallidicaule* Aellen | Kolano et al. (direct submission to Genbank); Fuentes-Bazan et al. (direct submission to Genbank) | | | | | KJ629055 | ** | HE855654 |
| *Dysphania ambrosioides* (L.) Mosyakin & Clemants | Kadereit et al. (direct submission to Genbank); Phan et al. (2021); Xu et al. (2017) | | | | | MK802956 | LC593165 | MF159497 |
| *Dysphania botrys* (L.) Mosyakin & Clemants | Kadereit et al. (direct submission to Genbank); Kadereit et al. (2003); Muller and Borsch (direct submission to Genbank) | | | | | MK802964 | AY270080 | AY514835 |
| *Dysphania graveolens* Mosyakin & Clemants | Fuentes-Bazan et al. (direct submission to Genbank) | | | | | HE855676 | ** | HE855609 |
| *Dysphania multifida* (L.) Mosyakin & Clemants | Kadereit et al. (direct submission to Genbank) | | | | | MK803003 | ** | ** |
| *Dysphania pumilio* (R. Br.) Mosyakin & Clemants | Kadereit et al. (direct submission to Genbank); Phan et al. (direct submission to Genbank); Fuentes-Bazan et al. (direct submission to Genbank) | | | | | MK803016 | LC593164 | HE855606 |
| *Dysphania schraderiana* (Schult.) Mosyakin & Clemants | Fuentes-Bazan et al. (2012) | | | | | HE577349 | ** | ** |
| *Exomis microphylla* (Thunb.) Aellen | Kadereit et al. (2010); Bezeng et al. (direct submission to Genbank) | | | | | HM587569 | HM587601 | JQ412243 |
| *Halimione pedunculata* (L.) Aellen | Kadereit et al. (2010); Kadereit et al. (2003) | | | | | HM587573 | AY270093 | ** |
| *Halimione portulacoides* (L.) Aellen | Kadereit et al. (2010); Pearse et al. (2015); Pagliano et al. (direct submission to Genbank) | | | | | HM587574 | KM360659 | DQ468648 |
| *Halimione verrucifera* (M. Bieb.) Aellen | Kadereit et al. (2010) | | | | | HM587575 | HM587606 | ** |
| *Holmbergia tweedii* (Moq.) Speg. | Zacharias and Baldwin (2010); Kadereit et al. (2003) | | | | | HM005842 | AY270100 | HM005761 |
| *Krascheninnikovia ceratoides* (L.) Gueldenst. | Seidl (direct submission to Genbank); Kadereit et al. (2003); Zhang et al. (direct submission to Genbank) | | | | | LR537419 | AY270105 | MK954301 |
| *Krascheninnikovia ceratoides* subsp. *lanata* (Pursh) Heklau | Heklau and Roser (2008); Ng et al. (direct submission to Genbank); Thornhill et al. (direct submission to Genbank) | | | | | AM849242 | KY584325 | MF963510 |
| *Microgynoecium tibeticum* Hook. f. | Fuentes-Bazan et al. (2012); Kadereit et al. (2003); Fuentes-Bazan et al. (direct submission to Genbank) | | | | | HE577363 | AY270107 | HE855639 |
| *Oxybasis glauca* (L.) S. Fuentes | Yao et al. (2017); Xu et al. (2017) | | | | | MF063412 | MF135443 | MF063946 |
| *Oxybasis rubra* Kar. & Kir. | Fuentes-Bazan et al. (2012); de Vere et al. (direct submission to Genbank); Fuentes-Bazan et al. (direct submission to Genbank) | | | | | HE577385 | JN892909 | HE855626 |
| *Oxybasis urbica* (L.) S. Fuentes | Fuentes-Bazan et al. (2012); Kadereit et al. (2010); Fuentes-Bazan et al. (direct submission to Genbank) | | | | | HE577384 | HM587596 | HE855630 |
| *Spinacia oleracea* L. | Li and Guy (direct submission to Genbank); Lu et al. (2019) | | | | | AF062088 | ** | MK090000 |
| *Spinacia tetrandra* Steven ex M. Bieb. | Fujito et al. (2015); Fuentes-Bazan et al. (direct submission to Genbank) | | | | | AB935687 | ** | HE855619 |
| *Spinacia turkestanica* Iljin | Fujito et al. (2015); Fuentes-Bazan et al. (direct submission to Genbank) | | | | | AB935684 | ** | HE855620 |
| *Stutzia dioica* (Nutt.) E. H. Zacharias | Fuentes-Bazan et al. (2012) | | | | | HE577362 | ** | ** |
| *Teloxys aristata* (L.) Moq. | Kadereit et al. (direct submission to Genbank); Kadereit et al. (2003); Fuentes-Bazan et al. (direct submission to Genbank) | | | | | MK803044 | AY270140 | HE855612 |
| *Teloxys aristata* (L.) Moq. | Li (direct submission to Genbank) | | | | | MW725199 | ** | ** |
| **Salsoloideae** |  |  |  |  |  |  |  |  |
| *Anabasis aphylla* L. | Akhani et al. (2007); Wen et al. (2010); Zhang et al. (direct submission to Genbank) | | | | | EF453380 | HM131746 | MK954240 |
| *Anabasis brevifolia* C. A. Mey. | Wen et al. (2010) | | | | | HM131610 | HM131747 | ** |
| *Girgensohnia oppositiflora* (Pall.) Fenzl | Wen et al. (2010); Kapralov et al. (2006) | | | | | HM131626 | HM131761 | DQ499405 |
| *Micropeplis arachnoidea* (Moq.) Bunge | Wen et al. (2010); Kadereit et al. (2017) | | | | | HM131630 | HM131764 | KY941481 |
| *Halogeton glomeratus* (M. Bieb.) Ledeb. | Wen et al. (2010); Kadereit et al. (2017) | | | | | HM131631 | HM131765 | KY941492 |
| *Halothamnus bottae* Jaub. & Spach | Khan et al. (direct submission to Genbank); Wen et al. (2010) | | | | | KF805127 | HM630097 | ** |
| *Halothamnus iliensis* (Lipsky) Botsch. | Schussler et al. (2017); | | | | | KX262607 | ** | ** |
| *Haloxylon ammodendron* (C. A. Mey.) Bunge ex Fenzl | Wen et al. (2010); Zhang et al. (direct submission to Genbank) | | | | | HM131632 | HM131766 | MK954259 |
| *Iljinia regelii* (Bunge) Korovin | Wen et al. (2010) | | | | | HM131635 | HM131769 | ** |
| *Noaea minuta* Boiss. & Balansa | Schussler et al. (2017) | | | | | KX262557 | ** | ** |
| *‘Oreosalsola' laricifolia* (Turcz. ex Litw.) Akhani | Wen et al. (2014); Wen et al. (2010) | | | | | KC310721 | HM131787 | KX133183 |
| *Salsola collina* Pall. | Wen et al. (2010); Xu et al. (direct submission to Genbank) | | | | | HM131648 | HM131782 | MH659995 |
| *Soda foliosa* (L.) Schrad. | Wen et al. (2010); Kadereit et al. (2017) | | | | | HM131651 | HM131784 | KY941494 |
| **Salicornioideae** |  |  |  |  |  |  |  |  |
| *Arthrocaulon macrostachyum* (Moric.) Piirainen & G. Kadereit | *Piirainen* et al. (2017); Kadereit et al. (2003); Pagliano et al. (direct submission to Genbank) | | | | | KU975174 | AY270058 | DQ465003 |
| *Tecticornia indica* (Willd.) K. A. Sheph. & Paul G.Wilson | Kadereit et al. (2005); Kadereit et al. (2003) | | | | | AY489243 | AY270096 | ** |
| *Kalidium foliatum* (Pall.) Moq. | Liang and Wu (2017); Wen et al. (2010) | | | | | KX133073 | HM131772 | KX133157 |
| *Salicornia europaea* L. | Kadereit et al. (2005); Wen et al. (2010); Yao et al. (2017) | | | | | AY489247 | HM131777 | MF063991 |
| *Salicornia fruticosa* (L.) L. | Kadereit et al. (2006); Pagliano et al. (direct submission to Genbank) | | | | | DQ340164 | ** | DQ468645 |
| **Suaedoideae** |  |  |  |  |  |  |  |  |
| *Suaeda aralocaspica* (Bunge) Freitag & Schütze | Wen et al. (2010); Rosnow et al. (2015); Kapralov et al. (2006) | | | | | HM131616 | KR057190 | DQ499411 |
| *Suaeda microphylla* Pall. | Wen et al. (2010) | | | | | HM131671 | HM131798 | ** |
| *Suaeda paradoxa* (Bunge) Bunge | Wen et al. (2010) | | | | | HM131672 | HM131799 | ** |
| *Suaeda physophora* Pall. | Wen et al. (2010) | | | | | HM131673 | HM131800 | ** |
| *Suaeda prostrata* Pall. | Wen et al. (2010) | | | | | HM131674 | HM131801 | ** |
| **Amaranthaceae** |  |  |  |  |  |  |  |  |
| *Alternanthera brasiliana* (L.) Kuntze | Sanchez-del Pino et al. (2012); Elansary et al. (2017) | | | | | JQ403565 | KX783819 | KX783628 |
| *Alternanthera caracasana* Kunth | Thenuwara Hannadige et al. (direct submission to Genbank); Schaefer et al. (2011); Muller and Borsch (direct submission to Genbank) | | | | | MK744122 | HM849761 | AY514794 |
| *Amaranthus hybridus* L. | Vorster and Simelane (direct submission to Genbank); Xu et al. (2017); Waselkov et al. (2018) | | | | | MT811924 | MF135436 | MG685142 |
| *Amaranthus spinosus* L. | Ranaweera et al. (direct submission to Genbank); Xu et al. (2017); Waselkov et al. (2018) | | | | | MN103757 | MF135474 | MG685172 |
| *Celosia trigyna* L. | Beard and Lawton-Rauh (direct submission to Genbank); Kapralov and Filatov (direct submission to Genbank); Muller and Borsch (direct submission to Genbank) | | | | | KC747456 | HQ237459 | AY514811 |
| *Iresine diffusa* Humb. & Bonpl. ex Willd. | Gruenstaeudl (direct submission to Genbank); International Barcode of Life (direct submission to Genbank); | | | | | LS452991 | JQ590121 | LS451274 |
| *Iresine palmeri* (S. Watson) Standl. | Gruenstaeudl (direct submission to Genbank); Kadereit et al. (2003); Mueller and Borsch (2005) | | | | | LS452953 | AY270101 | AY514804 |
| *Polycnemum majus* A. Braun | Mueller and Borsch (2005) | | | | | ** | AY270118 | AY514839 |
| **Outgroups** |  |  |  |  |  |  |  |  |
| *Phaulothamnus spinescens* A. Gray | Manhart and Rettig (direct submission to Genbank); Mueller and Borsch (2005) | | | | | ** | M97887 | AY514846 |
| *Rhabdodendron amazonicum* (Spruce ex Benth.) Huber | Brockington et al. (2008); Fay et al. (1997); Crawley and Hilu (2011) | | | | | EU410359 | Z97649 | JQ844136 |
| *Simmondsia chinensis* (Link) C. K. Schneid. | Hoot et al. (1999); Mueller and Borsch (direct submission to Genbank) | | | | | ** | AF093732 | AY514854 |

**, missing from NCBI database
