# Supplementary material for: Phylogenetic placement of the monotypic Baolia (Amaranthaceae s.l.) based on morphological and molecular evidence
Source: BMC Plant Biol. 2024 May 25;24:456. doi: 10.1186/s12870-024-05164-8 (PMC11127444; doi:10.1186/s12870-024-05164-8)
Supplement: Supplementary file 2 — Supplementary Material 2. [file 12870_2024_5164_MOESM2_ESM.zip › Table S7_Coded matrix for Baolia, Acroglochin, and Corispermoideae for ancestral character state reconstructions.docx]

**Table S7 Coded matrix for *Baolia*, *Acroglochin*, and Corispermoideae for ancestral character state reconstructions**

| Taxon/character states | 1 | 2 | 3 | 4 | 5 | 6 | 7 | 8 | 9 | 10 |
| --- | --- | --- | --- | --- | --- | --- | --- | --- | --- | --- |
| *Acroglochin persicarioides* | 0 | 1 | 0 | 0 | 2 | 1 | 0 | 0 | 0 | 1 |
| *Baolia bracteata* | 0 | 0 | 2 | 1 | 2 | 0 | 2 | 0 | 1 | 0 |
| **Corispermoideae** |  |  |  |  |  |  |  |  |  |  |
| *Agriophyllum pungens* | 3 | 0 | 1 | 0 | 0 | 1 | 0 | 1 | 1 | 0 |
| *Anthochlamys multinervis* | 1 | 0 | 1 | 0 | 1 | 0 | 1 | 1 | 1 | 0 |
| *Corispermum americanum* | 2 | 0 | 1 | 0 | 0 | 0 | 0 | 1 | 1 | 0 |
| *Corispermum chinganicum* | 2 | 0 | 1 | 0 | 0 | 0 | 0 | 1 | 1 | 0 |
| *Corispermum filifolium* | 2 | 0 | 1 | 0 | 0 | 0 | 0 | 1 | 1 | 0 |
| *Corispermum puberulum* | 2 | 0 | 1 | 0 | 0 | 0 | 0 | 1 | 1 | 0 |
| **Chenopodioideae** |  |  |  |  |  |  |  |  |  |  |
| *Archiatriplex nanpinensis* | 0 | 0 | 2 | 0 | 6 | 0 | 1 | 0 | 0 | 1 |
| *Atriplex hortensis, A. sagittata* | 4 | 0 | 2 | 0 | 4 | 0 | 1 | 0 | 0 | 2 |
| *Axyris* (different species) | 6 | 0 | 2 | 0 | 1 | 0 | 0 | 0 | 1 | 1 |
| *Blitum* (different species) | 4 | 0 | 2 | 0 | 3 | 0 | 0 | 0 | 0 | 1 |
| *Ceratocarpus arenarius* | 6 | 0 | 0 | 1 | 0 | 0 | 0 | 0 | 0 | 0 |
| *Chenopodiastrum hybridum* | 4 | 0 | 2 | 0 | 2 | 0 | 1 | 0 | 0 | 1 |
| *Chenopodium* (different species) | 4 | 0 | 2 | 0 | 2 | 0 | 1 | 0 | 0 | 1 |
| *Dysphania ambrosioides, D. multifida* | 5 | 0 | 2 | 0 | 2 | 0 | 3 | 0 | 0 | 1 |
| *Dysphania botrys, D. graveolens, D. pumilio, D. schraderiana* | 5 | 0 | 2 | 0 | 2 | 0 | 1 | 0 | 0 | 1 |
| *Exomis microphylla* | 4 | 0 | 2 | 0 | 0 | 0 | 3 | 0 | 0 | 1 |
| *Halimione* (different species) | 4 | 0 | 2 | 0 | 4 | 0 | 0 | 0 | 0 | 0 |
| *Holmbergia tweedii* | 4 | 0 | 2 | 0 | 2 | 0 | 0 | 0 | 0 | 1 |
| *Krascheninnikovia ceratoides* | 6 | 0 | 2 | 0 | 4 | 0 | 4 | 0 | 0 | 0 |
| *Microgynoecium tibeticum* | 4 | 0 | 2 | 0 | 0 | 0 | 1 | 0 | 0 | 1 |
| *Oxybasis glauca* | 4 | 0 | 2 | 0 | 6 | 2 | 0 | 0 | 0 | 1 |
| *Oxybasis rubra* | 4 | 0 | 2 | 0 | 5 | 2 | 1 | 0 | 0 | 2 |
| *Oxybasis urbica* | 4 | 0 | 2 | 0 | 2 | 0 | 1 | 0 | 0 | 2 |
| *Spinacia* (different species) | 4 | 0 | 2 | 0 | 2 | 0 | 0 | 0 | 0 | 0 |
| *Teloxys aristata* | 0 | 1 | 0 | 0 | 2 | 0 | 0 | 0 | 0 | 1 |
| **Outgroups** (selected) |  |  |  |  |  |  |  |  |  |  |
| *Arthrocaulon macrostachyum* | 0 | 0 | 2 | 0 | 3 | 0 | 0 | 0 | 0 | 1 |
| *Bassia prostrata* | 1 | 0 | 2 | 0 | 2 | 0 | 0 | 0 | 0 | 0 |
| *Girgensohnia oppositifolia* | 0 | 0 | 2 | 1 | 2 | 0 | 1 | 0 | 0 | 0 |
| *Hablitzia tamnoides* | 0 | 0 | 2 | 0 | 2 | 1 | 0 | 0 | 0 | 1 |
| *Halothamnus iliensis* | 0 | 0 | 2 | 1 | 2 | 0 | 0 | 0 | 1 | 0 |
| *Salicornia fruticosa* | 0 | 0 | 2 | 0 | 3 | 2 | 0 | 0 | 0 | 0 |
| *Soda foliosa* | 0 | 0 | 2 | 1 | 2 | 0 | 0 | 0 | 0 | 0 |
| *Suaeda prostrata* | 0 | 0 | 2 | 1 | 2 | 2 | 0 | 0 | 0 | 2 |

**Note:** 1. Hairs on stems and leaves: 0 – absent or papillae; 1 – mostly simple curved hairs; 2 – branched hairs; 3 – dendroid hairs; 4 – prevailing bladder hairs; 5 – simple, glandular hairs and subsessile glands; 6 – prevailing stellate hairs.

2. Acicular apices: 0 – absent; 1 – present.

3. Inflorescence: 0 – monochasium; 1 – spikes; 2 – composed of clusters.

4. Bracteoles: 0 – absent; 1 – present.

5. Perianth: 0 – absent or reduced (1–2 segments) and hyaline; 1 – five hyaline segments; 2 – (4)5 green segments; 3 – three or four green segments usually [turning] fleshy (outgroups); 4 – of two accrescent segments forming bract-like cover and five green segments; 5 – three to five hyaline segments within one individuum; 6 – three to four green segments (at least in female flowers).

6. Fruit: 0 – indehiscent; 1 – dehiscent by a lid; 2 – irregularly dehiscent.

7. Pericarp surface: 0 – smooth; 1 – papillate or mamillate (sometimes with trichomes) with non-bursting outer walls of the exocarp cells; 2 – papillate with bursting outer walls of the exocarp cells and forming at fruiting honey-comb sculpture; 3 – with bladder hairs; 4 – with stellate hairs.

8. Pericarp wing: 0 – absent; 1 – marginally present.

9. Sclerenchymatous tissue in the pericarp: 0 – absent; 1 – present.

10. Seed-coat testa: 0 – very thin (up to 8 µm); 1 – more than 10 µm; 2 – both types 0 & 1 (heterospermic).
