# Supplementary material for: Phylogenetic placement of the monotypic Baolia (Amaranthaceae s.l.) based on morphological and molecular evidence
Source: BMC Plant Biol. 2024 May 25;24:456. doi: 10.1186/s12870-024-05164-8 (PMC11127444; doi:10.1186/s12870-024-05164-8)
Supplement: Supplementary file 1 — Supplementary Material 1. [file Fig. S2_Sequence identity plot comparing the 11 chloroplast genomes with Baolia bracteata as a reference by using mVISTA.pdf › Fig. S2_Sequence identity plot comparing the 11 chloroplast genomes with Baolia bracteata as a reference by using mVISTA.pdf]

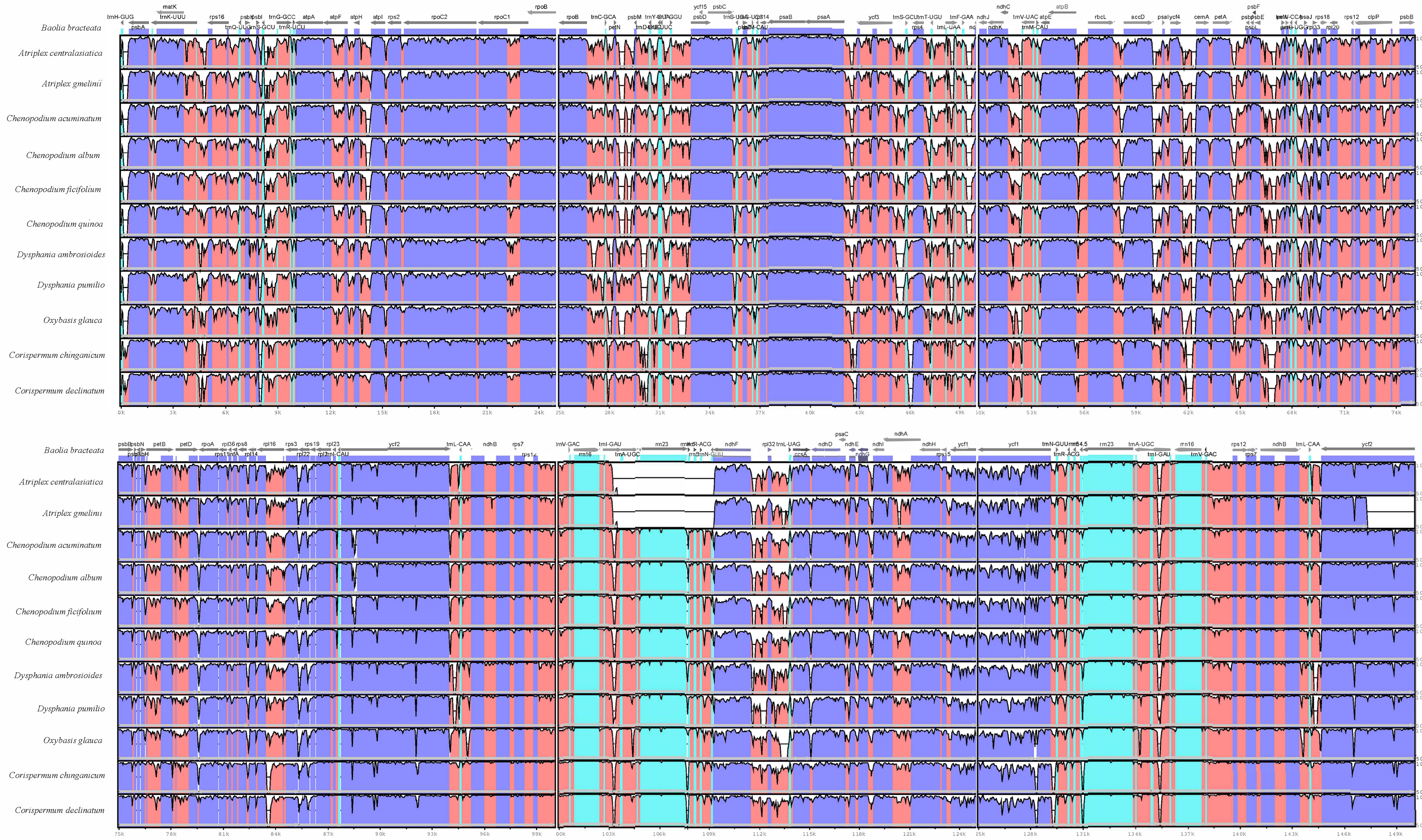

Sequence identity plot comparing the 11 chloroplast genomes with *Baolia bracteata* as a reference by using mVISTA. Gray arrows and thick black lines above the alignment indicate genes with their orientation and the position of the inverted repeats (IRs), respectively. A cut-off of 70% identity was used for the plots, and the Y-scale represents the percent identity ranging from 50 to 100%.
